# Efficacy and safety of human papillomavirus vaccination in HIV-infected patients: a systematic review and meta-analysis

**DOI:** 10.1038/s41598-021-83727-7

**Published:** 2021-03-02

**Authors:** Antonella Zizza, Federico Banchelli, Marcello Guido, Claudia Marotta, Francesco Di Gennaro, Walter Mazzucco, Vanna Pistotti, Roberto D’Amico

**Affiliations:** 1 Institute of Clinical Physiology, National Research Council, 73100 Lecce, Italy; 2 Department of Medical and Surgical Sciences, University of Modena and Reggio Emilia, 41100 Modena, Italy; 3 Laboratory of Hygiene, Department of Biological and Environmental Sciences and Technologies, University of Salento, Via Prov.Le Lecce-Monteroni, 165, 73100 Lecce, Italy; 4 Inter-University Centre of Research on Influenza and Other Transmissible Infections (CIRI-IT), University of Genoa, 16100 Genoa, Italy; 5 Department of Health Promotion, Maternal and Infant Care, Internal Medicine and Medical Specialties (PROMISE), University of Palermo, 90100 Palermo, Italy; 6 Medical Direction, IRCCS Neuromed, 86170 Pozzilli, IS Italy; 7 Department of Infectious Diseases, University of Bari “Aldo Moro”, 70124 Bari, Italy; 8 Clinical Epidemiology and Cancer Registry Unit, COVID-19 Sicilian Regional Reference Lab, Palermo University Hospital (AOUP) “P. Giaccone”, 90100 Palermo, Italy; 9 Division of Biostatistics and Epidemiology, Department of Pediatric, Cincinnati College of Medicine, Cincinnati Children’s Hospital Medical Center, Cincinnati, OH USA; 10 Indipendent Researcher, Milan, Italy

**Keywords:** Health care, Disease prevention, Public health

## Abstract

The prophylactic vaccines available to protect against infections by HPV are well tolerated and highly immunogenic. People with HIV have a higher risk of developing HPV infection and HPV-associated cancers due to a lower immune response, and due to viral interactions. We performed a systematic review of RCTs to assess HPV vaccines efficacy and safety on HIV-infected people compared to placebo or no intervention in terms of seroconversion, infections, neoplasms, adverse events, CD4+ T-cell count and HIV viral load. The vaccine-group showed a seroconversion rate close to 100% for each vaccine and a significantly higher level of antibodies against HPV vaccine types, as compared to the placebo group (MD = 4333.3, 95% CI 2701.4; 5965.1 GMT EL.U./ml for HPV type 16 and MD = 1408.8, 95% CI 414.8; 2394.7 GMT EL.U./ml for HPV type 18). There were also no differences in terms of severe adverse events (RR = 0.6, 95% CI 0.2; 1.6) and no severe adverse events (RR = 0.6, 95% CI 0.9; 1.2) between vaccine and placebo groups. Secondary outcomes, such as CD4 + T-cell count and HIV viral load, did not differ between groups (MD = 14.8, 95% CI − 35.1; 64.6 cells/µl and MD = 0.0, 95% CI − 0.3; 0.3 log10 RNA copies/ml, respectively). Information on the remaining outcomes was scarce and that did not allow us to combine the data. The results support the use of the HPV vaccine in HIV-infected patients and highlight the need of further RCTs assessing the effectiveness of the HPV vaccine on infections and neoplasms.

## Introduction

### Description of the condition

Human papillomaviruses (HPV) are considered the most common causative agents of sexually transmitted diseases in the world^1^. So far, over 200 virus types have been identified and of these, more than 40 types are sexually transmitted and can cause premalignant and malignant lesions of the cervix, anal, vulvar, vaginal, penile, oropharyngeal cancers as well as genital warts. Of these, HPV types 16, 18, 31, 33, 35, 39, 45, 51, 52, 56, 58, 59, 66 and 68 have been classified as oncogenic and HPV types 26, 30, 34, 53, 67, 69, 70, 73, 82 and 97 as probably/possibly carcinogenic. They are all defined as high risk. Types 16 and 18 cause 62.6% and 15.7% of cervical cancers worldwide respectively^2,3^. The HPV types classified as non-oncogenic (or low risk) include 6, 11, 32, 40, 42, 43, 44, 54, 55, 57, 61, 62, 64, 71, 72, 74, 81, 83, 84, 89, 90, 91 and they can develop benign tumours such as genital warts and respiratory papillomatosis. Among these, HPV 6 and 11 are the most frequent types^4,5^.

Cervical cancer is the fourth most common cancer in women with estimated 528,000 new cases and 266,000 deaths annually^6^. Individuals infected with human immunodeficiency virus (HIV) have a higher risk of developing HPV incident and persistent infection, precancerous lesions and HPV-related cancers due to a lower immunological response, and due to viral interactions^7,8^. Each infection favours the acquisition and the amplification of the other ones^9^. HIV promotes initial infection through the destruction of the epithelial tight junctions^10^. People with HIV infections have a more rapid progression to malignancy^11,12^. Men who have Sex with Men (MSM) is the population with the highest risk of HPV anal infection^13^. Anal HPV infection, which contributes to the development of anal warts and anal cancers, is frequent amongst HIV + MSM, with risk of invasive anal cancer 37-fold greater than HIV + ^14^.

Furthermore, HIV may reduce responsiveness to vaccines and their effectiveness due to immune deficits as the infection advances^15,16^.

### Description of the intervention

Three types of prophylactic vaccines composed of virus-like particles are currently available: the bivalent (2vHPV, Cervarix, GLAXOSMITH-KLINE, Brentford, England), the quadrivalent (4vHPV, Gardasil, MERCK & CO., Inc., Kenilworth, NJ) and the nonavalent (9vHPV, Gardasil 9, MERCK & CO., Inc., Kenilworth, NJ) vaccines to prevent cervical cancer. The 2vHPV and 4vHPV ones are recombinant vaccines that protect against HPV types 16 and 18 persistent infections. In addition, the 4vHPV protects against HPV types 6 and 11 infections. Cervarix contains AS04, an adjuvant system that elicits a protective immune response for a longer period of time. 9vHPV vaccine to prevent infections and diseases related to nine HPV types (6, 11, 16, 18, 31, 33, 45, 52, 58).

Cervarix was licensed in Australia, Philippines, EU in 2007, while in USA in 2009 and Gardasil was licensed in EU in 2007. The latter (Gardasil 9) was licensed in the USA in 2014, in Canada, in the EU and in Australia in 2015 and in other countries between 2015 and 2016.

### Why it is important to do this review?

The body of evidence suggests that HPV vaccines are highly immunogenic and well tolerated and their immune response persists up to 10 years after vaccination. They induce excellent protection against vaccine-included genotypes, anogenital warts and high-grade intraepithelial neoplasia.

All HPV vaccines are generally well tolerated with mild or self-limited injection site reactions being the most common adverse events. No serious effects have been found to be related to vaccination.

Several studies have described the impact and effectiveness of HPV vaccination, once it has been included in public health programs^17–20^. Prophylactic HPV vaccines may also benefit HIV + patients, however, little is known about their safety and efficacy in this population.

### Objectives

This review seeks to evaluate the efficacy and safety of vaccines to prevent HPV infections and correlated lesions in cervical, vulvar, vaginal, penile, anal or oral districts in HIV-infected patients by meta-analysing the results of randomised controlled trials (RCTs).

## Methods

This systematic literature review was performed following the guidelines of the PRISMA Statement^21^ and of the Cochrane Handbook for Systematic Reviews of Interventions^22^. The full research protocol is available on Prospero Center for Reviews and Dissemination (PROSPERO) under the registration number of CRD42018084619. Ethical committee approval was not requested because this review used only published data.

### Search strategy

#### Database search

A comprehensive search strategy of all relevant studies regardless of language or publication status (published, unpublished, in press and in progress) was performed on the following electronic databases: EMBASE, PubMed (Medline) and Cochrane Database of Systematic Reviews up to 22-nd May, 2019. Details on the search strategy are reported in Supplemental Table S1.

#### Other sources

The reference lists of the identified studies were also checked for additional studies. The clinical trials registry platform ClinicalTrials.gov was also searched for any ongoing trial.

### Inclusion criteria

RCTs that compared the use of 2vHPV, 4vHPV or 9vHPV vaccines for HPV prevention against placebo or no intervention in HIV + individuals were evaluated for inclusion. Quasi-randomized trials and nonrandomized studies were not included.

### Study outcomes

#### Primary outcomes

Efficacy:

- HPV seroconversion:
  o for HPV types 6, 11, 16, 18, 31, 33, 45, 52, 58.
- Presence of infection:
  o due to HPV types 6, 11, 16, 18, 31, 33, 45, 52, 58;
  o in the cervical, vulvar, vaginal, penile, anal or oral districts.
- Presence of grades 2 or 3 neoplasia:
  o Cervical Intraepithelial Neoplasia (CIN);
  o Vaginal Intraepithelial Neoplasia (VAIN);
  o Vulvar Intraepithelial Neoplasia (VIN);
  o Anal Intraepithelial Neoplasia (AIN);
  o Penile Intraepithelial Neoplasia (PeIN);
  o Oral Intraepithelial Neoplasia (OIN).

Safety:

- Adverse events (AEs);
- Mortality for all causes.

#### Secondary outcomes

- HIV viral load (VL);
- CD4 + T-cell count;
- Completion rate of three doses of HPV vaccination series.

### Selection of studies and data extraction

After the removal of duplicates, the records were independently screened according to pre-specified criteria by four review authors (FDG, MG, CM, AZ) in two separate stages. First, titles, abstracts and descriptor terms were screened for eligibility followed by the full text. Disagreements on eligibility amongst the reviewers were resolved by discussion. Then, data was independently extracted, using a data extraction form, by four reviewers (FDG, MG, CM, AZ). Disagreements were solved by discussion amongst authors. The sources for data extraction were the original articles and the ClinicalTrials.gov registry.

Data on study characteristics (authors, year, country and study design), patient features (number of analysed and randomised patients, age, gender, risk factors), baseline characteristics (CD4 + T-cell count and VL) and peculiarity of interventions (type of vaccine, number of doses and timing, description of placebo) was extracted from each study. Primary outcomes of efficacy (HPV seroconversion, immunogenicity, presence of infection and lesion) and safety (all-causes mortality and AEs), as well as secondary outcomes (full vaccination course, CD4 + T-cell count and HIV VL after the vaccination) and the numbers of patients that were analysed by the authors, were also extracted.

### Assessment of risk of bias in included studies

The Cochrane Risk of Bias (RoB) tool was used^22^, which includes the following criteria: random sequence generation (selection bias), allocation concealment (selection bias), blinding of participants and outcome assessors (performance and detection biases), incomplete outcome data (attrition bias), selective outcome reporting (reporting bias) and other biases. The RoB of each study was explicitly judged on each criterion and classified as ’low’, ’high’, or ’unclear’. An unclear RoB resulted from a lack of sufficient information about methods used in studies, making it impossible to judge whether or not the bias was present. In this review, risk of attrition bias was judged as ’low risk’ when the percentage of participants with missing data was low for primary outcomes, which was arbitrarily set at values lower than 15%, and when numbers and causes of losses to follow-up were balanced between arms.

To summarize the overall RoB for a study, random sequence generation and allocation concealment, blinding of outcome assessors and incomplete outcome data were considered in order to classify each study as: ’low risk of bias’ when all four criteria were met; ’high risk of bias’ when all criteria were unmet; ’moderate risk of bias’ in the remaining cases.

We also assessed RoB for AEs. We evaluated methods for monitoring and detecting an AE for each study: ‘low risk of bias for AEs’ was assigned when authors actively monitored for AEs, whereas a ‘high risk of bias’ was assigned in the case they simply provided spontaneous reporting of the occurring AEs. We also assessed whether the authors defined severe and not severe AEs according to a recognised international classification. The RoB of each study was assessed and discussed by all reviewers.

### Statistical analysis and data synthesis

Our unit of data extraction, evaluation, and analysis was the primary randomized trial. The association between the intervention and dichotomous outcomes was measured by using the Risk Ratio (RR) with 95% confidence interval (95% CI), whereas for continuous variables it was measured by using the mean difference (MD) with 95% CI. In calculating the MD, equality of variances between the two groups was considered, unless evidence of not equal variances was present. All association measures were calculated considering the placebo group as the reference category. Meta-analyses were performed for all outcomes, provided that at least two studies were available, using a random effects model^23^. The pooling method was inverse variance (IV) weighting for quantitative outcomes and Mantel–Haenszel (MH) weighting for dichotomous variables. For dichotomous outcomes, only studies with at least one observed event were considered for data synthesis. The presence of statistical heterogeneity was assessed by Tau^2^ and I^2^ statistics. Data synthesis was not performed when I^2^ statistics > 90%.

Statistical analyses were carried out with R 3.6.3 statistical software (The R Foundation for Statistical Computing, Wien) at p < 0.05 significance level.

### Subgroup analysis

The effect of vaccines in the following subgroups were also sought:

- Gender (female, male);
- Age (children, young women, adults);
- Presence of risk factors (e.g. MSM);
- CD4 + T-cell count (low, high);
- HIV VL (suppressed, not suppressed).

### Summary of findings

The summary of findings was reported following the GRADE criteria^24^ and was explicitly rated for each outcome on a four-levels scale (‘high’, ’moderate’, ‘low’ or ‘very low’ certainty). Since we have included only RCTs in this review, our rating started from a high level of certainty and then we eventually downgraded it by considering the following criteria: RoB of included studies, inconsistency and imprecision of relative effect estimates, indirectness of evidence and publication bias.

## Results

### Selection of studies

**Figure 1:**
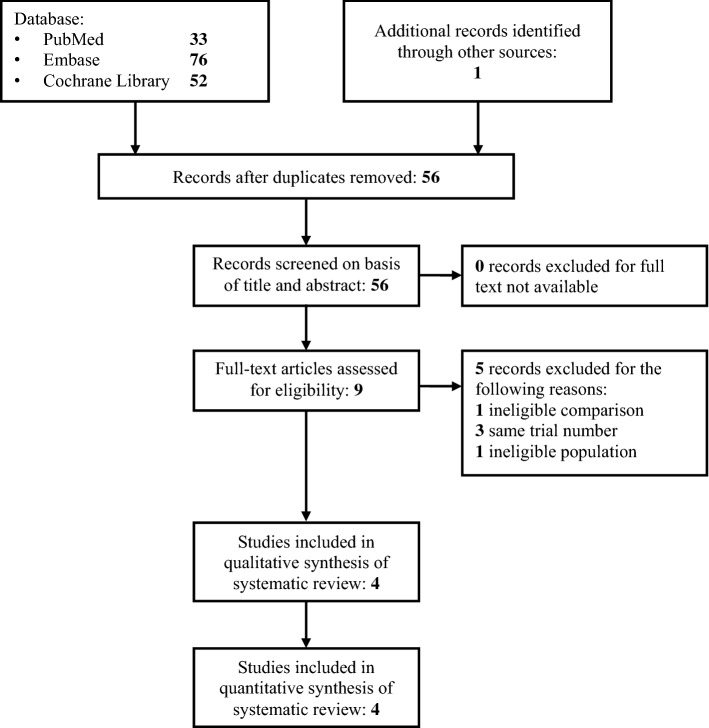
Flow chart of primary studies selection.

There were fifty-six studies that were identified by our search strategy on 22-nd May, 2019. All of them were screened for inclusion by title and abstract assessment. Nine full-text articles were screened for eligibility and four of them met the inclusion criteria. A flow diagram describing the study selection process is reported in Fig. 1.

### Included studies

Four randomised placebo-controlled trials were included in this review^25–28^. The characteristics of these studies are reported in Supplemental Table S2.

### Population

The studies included people with HIV: women aged 18–25 years with mean age of vaccine group of 21.6 ± 2.21 years and placebo group of 22.7 ± 1.7^25^; MSM ≥ 18 years with mean age of vaccine group of 37.3 ± 10.6 years and control group of 40.5 ± 10.02^27^; children aged 7–12 years with mean age of vaccine group of 10 years (95% CI 9.7–10.3) and placebo of 9.9 years (95% CI 9.4–10.4)^26^; MSM and women ≥ 27 years with median age of vaccine group of 47 (IQR 40–52) and placebo of 48 (IQR 42–53)^28^.

The RCTs were carried out between 2008 and 2014 in South Africa, Spain, USA and Brazil. For one study the start and end dates were not reported^26^. The four studies randomised a total of 950 participants. Overall, the vaccine groups consisted of 511 HIV + patients, ranging between 61 and 288 participants per study. The placebo groups consisted of 439 HIV + patients, ranging between 59 and 287 per study.

### Intervention and comparator

Three of the studies assessed immunogenicity and safety of the 4vHPV^26–28^ and one evaluated the reactogenicity and safety of 2vHPV^25^. In one trial, participants have received the HPV-16/18 AS04-adjuvanted vaccine or the placebo at 0, 1, and 6 months^25^. In the other three trials, quadrivalent vaccine and placebo were administered at 0, 8, 24 weeks. None of the included studies reported the use of the 9vHPV.

### Outcomes

Outcomes in primary studies were reported at different time points as reported in Supplemental Table S3. Only time points up to 12 months after the onset of the vaccination series were considered.

Based on data availability, we decided to assess the outcomes at the following time points:

- Seroconversion outcomes were assessed after the end of the vaccination series (month 7^25,26^ and week 28^27^).
- Anal and oral infections were assessed as persistent infections during the study period, including single detection at the final visit^28^.
- AIN was assessed as High-Grade AIN on anal biopsy outcomes during the study period (“full ITT approach” as stated by the authors)^28^.
- Abnormal Anal Cytology was assessed after the end of the vaccination series (week 52)^28^.
- AEs outcomes were assessed after the end of the vaccination series (month 7^25^, month 6^26^, week 28^27^ and week 48^28^).
- All-cause mortality outcome was assessed after the end of the vaccination series (month 12^25^, month 6^26^ and week 48^28^).
- CD4+ T-cell count outcome was assessed after the 2-nd vaccination (month 2^25,26^, and week 8^27^) and after the end of the vaccination series (month 6^25,26^, and week 28^27^).
- HIV VL outcome was assessed after the 2-nd vaccination (month 2^25^ and week 8^27^) and after the end of the vaccination series (month 6^25^ and week 28^27^).

#### Seroconversion

The seroconversion outcome was reported in two different ways: (1) as a continuous variable that measured the number of serum antibodies against HPV, expressed as the Geometric Mean antibody Titre (GMT) of enzyme-linked immunosorbent assay units per ml (EL.U./ml); (2) as a dichotomous variable that described the presence of serum antibodies against HPV.

Considering the continuous measurement, two studies included data on seroconversion for both vaccine and placebo groups after the end of the vaccination series^25,27^. The data in Denny L et al. was available for HPV types 16 and 18 (2vHPV)^25^ whereas the data in Levin MJ et al. for HPV types 6, 11, 16, and 18 (4vHPV)^27^.

Considering the dichotomous measurement, two studies reported the data. HPV types 6 and 11 were reported in Levin MJ et al.^27^, whereas HPV types 16 and 18 were reported in both^25,27^.

The data reported in Levin MJ et al.^27^, did not include patients with protocol violations, unevaluable specimens, or the presence of type-specific antibodies at baseline^27^, whereas the data in Denny et al. were reported only for those subjects with available immunogenicity data at 7 months^25^.

No study assessed seroconversion for HPV types 31, 33, 45, 52 and 58.

#### Presence of infections

One study reported the presence of anal and oral infections assessed in single detection at final visit or as confirmed persistent infections of any of HPV types 6, 11, 16, or 18^28^. None of the included studies have reported results concerning the presence of infections by HPV types 6, 11, 16, 18, 31, 33, 45, 52, 58 in the cervical, vulvar, vaginal districts.

#### Presence of grade 2 or 3 neoplasia

One study reported data on high grade AIN and abnormal anal cytology^28^. None of the included studies reported results concerning the presence of grade 2 or 3 cervical, vaginal, vulvar, penile or oral intraepithelial neoplasia.

#### Mortality for all causes

There were three included studies that reported data on mortality for all causes after the vaccination series^25,26,28^.

#### Adverse events

Data on the number of patients with AEs for both vaccine and placebo groups after the end of the vaccination series were reported by all the four included studies. Overall, there was a high number of AEs for which data were reported. We decided to include in our review and data synthesis only the following two outcomes: 1) Severe AEs (SAEs); 2) not severe AEs.

Data on SAEs were reported by all four included studies and their definitions were similar.

Data on not severe AEs were reported in all four included studies and their definitions were similar^25–28^, but we did not consider the data by Wilkin TJ et al. because the reported time was not homogeneous with the other studies.

Two studies^27,28^ used a table of grading to describe the severity of AEs. In Denny et al.^25^, SAEs were defined as AEs prompting emergency room or physician visits that were not related to common diseases or routine visits for physical examination or vaccination and as SAEs that were not related to common diseases. Common diseases include upper respiratory infections, sinusitis, pharyngitis, gastroenteritis, urinary tract infections, cervico-vaginal yeast infections, menstrual cycle abnormalities. Instead, in Hidalgo-Tenorio C et al. a definition of AEs was not provided^26^.

Full details of AEs are reported in Supplemental Table S4.

#### *CD4* + *T-cell count*

There were three included studies that reported data on CD4 + T-cell count^25–28^ after the 2nd vaccination and after the end of the vaccination series. These data were reported only for those subjects with available CD4 + T-cell count results at the respective time points and were expressed using the same unit of measurement (number of CD4 + cells per µl).

#### HIV VL or shedding

There were two included studies that reported data on HIV VL or shedding^25,27^ after the 2nd vaccination and after the end of the vaccination series. These data were reported only for those subjects with available HIV VL results at the respective time points and were expressed using the same unit of measurement (log_10_ RNA copies/ml).

#### Completion rate

All the included studies reported data on the number of patients who completed the vaccination series.

### Issues on data extraction

Data on CD4 + T-cell count and HIV VL outcomes in Denny L et al. were reported as median and inter-quartile range (IQR)^25^. The mean was approximated as the central value of the IQR and the standard deviation was approximated as the IQR divided by 1.35, based on the assumption of a Gaussian distribution.

Data on CD4 + T-cell count and HIV VL outcomes in Hidalgo-Tenorio et al.^26^ were reported as group means and p-value from a t test for two independent samples. The standard deviations for the two groups were both set to the value of the pooled standard deviation.

Lower and upper limits of the confidence interval for HPV type 18 immunogenicity in Levin MJ et al.^27^ for the placebo group were the same, due to decimal approximations. In this case, we added 0.5 to the upper limit.

### Assessment of risk of bias

**Figure 2:**
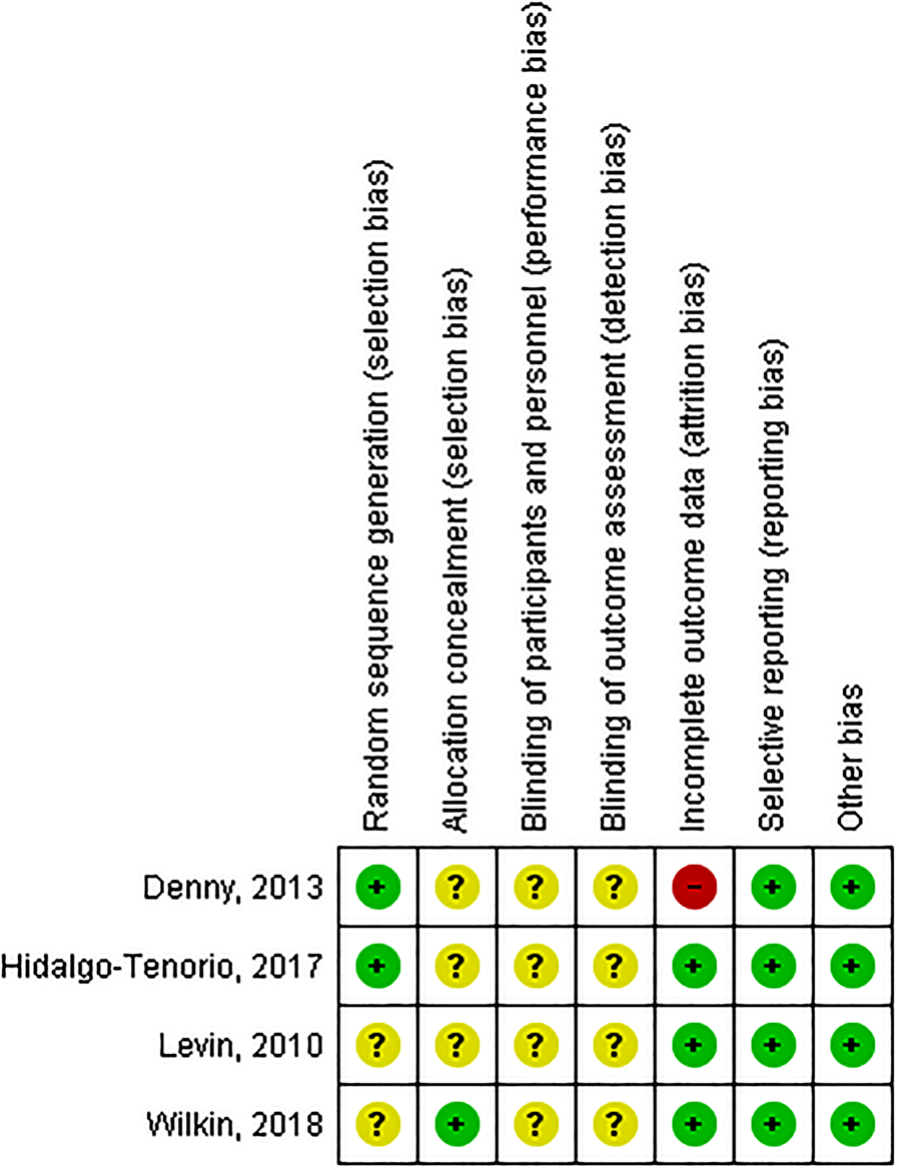
Risk of bias summary.

Assessment of RoB for each included study—using the Cochrane Collaboration criteria—is reported in Fig. 2. We rated two studies at low risk for random sequence generation^25,26^ and two at unclear risk^27,28^. RoB related to allocation concealment was judged unclear for three studies^25–27^ and low in the remaining one^28^. Performance bias (blinding of participants and personnel) and detection bias (blinding of outcome assessment) were rated at unclear risk for all the studies, since in all the RCTs the participants and the outcome assessors were blinded but none of the studies reported details on the methods of blinding. We found that three studies were at low risk of attrition bias, since participants with missing data were less than 15% and were balanced between arms. Conversely, one study^25^ was at high risk of attrition bias for the seroconversion outcome. Finally, as for the reporting bias and for other biases, we rated all studies at low risk.

Overall, we judged all studies to be at moderate RoB.

RoB for AEs was rated as low for all studies since all researchers provided active monitoring for AEs. Data on AEs were ranked by all studies according to a 4 levels severity scale. Two studies^27,28^ defined AEs and SAEs according to an accepted international classification (Division of AIDS Table for Grading the Severity of Adult AEs) whereas the remaining two studies measured AEs using an ad hoc developed questionnaire.

### Data synthesis

#### Seroconversion

**Figure 3:**
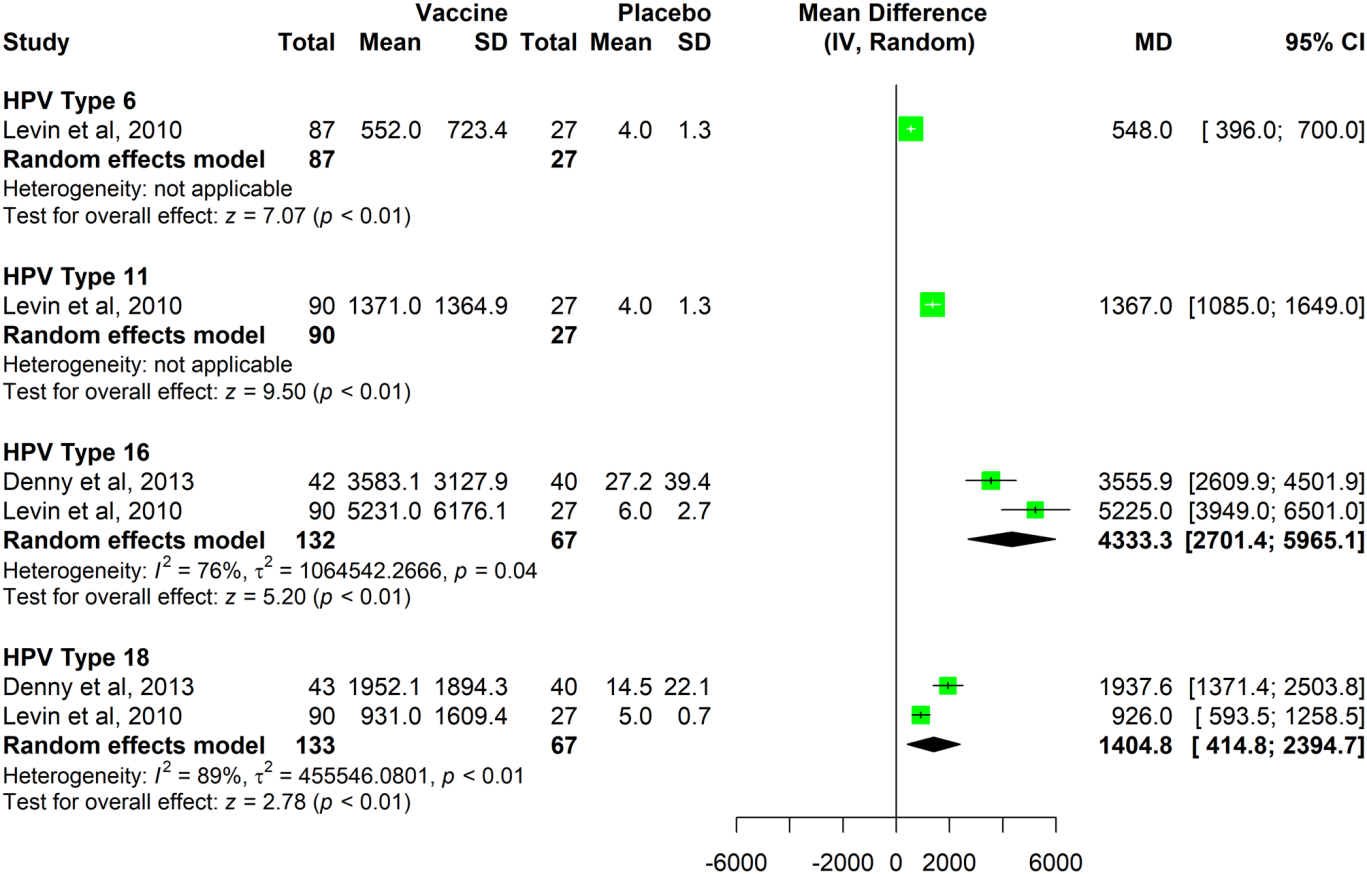
Data synthesis for seroconversion outcome (dichotomous).

The combined MDs in the immunogenicity outcome, considering unequal variances between groups, were 4333.3 GMT EL. U./ml (95% CI 2701.4–5965.1) for HPV 16 and 1404.8 GMT EL.U./ml (95% CI 414.8–2394.7) for HPV 18 (Fig. 3). We decided against presenting results of the meta-analysis for the dichotomous seroconversion outcome due to the high statistical heterogeneity observed across studies (Supplemental Fig. S1). One study^25^ comprised a population of women aged 18–25, whereas the other two studies^26,27^ comprised two populations of MSM and children aged 7–12 respectively.

#### Presence of infections or high-grade neoplasia

The results concerning the presence of anal and oral infections, as well as high grade AIN and abnormal anal cytology are reported in Supplemental Fig. S2. Data synthesis was not carried out since these outcomes were reported only in one study.

#### Safety outcomes

**Figure 4:**
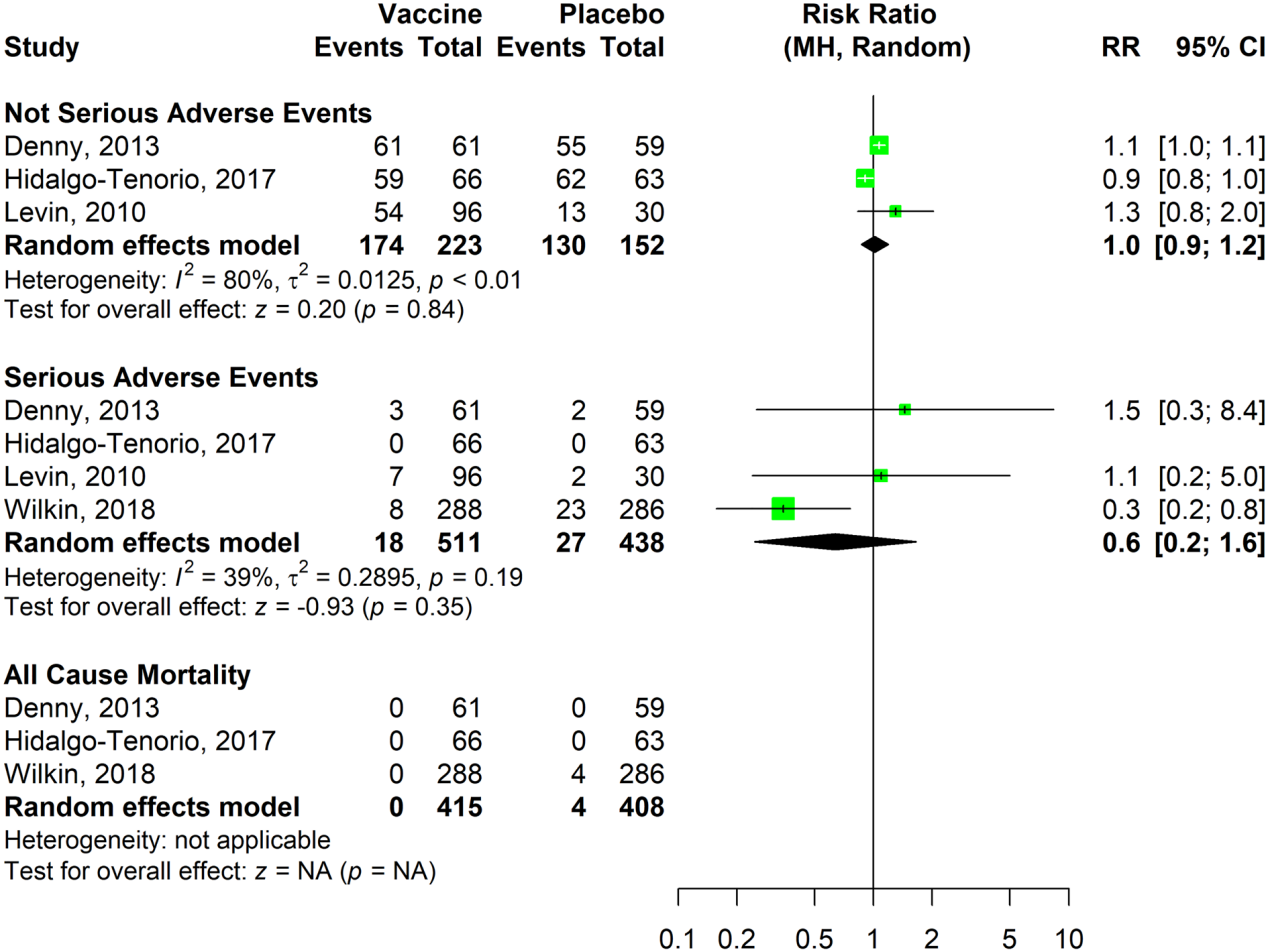
Data synthesis for seroconversion outcome (continuous).

The results concerning the AEs and mortality outcomes (dichotomous outcomes) are reported in Fig. 4. The combined RRs were 1.0 (95% CI 0.9–1.2) for not serious AEs and 0.6 (95% CI 0.2–1.6) for SAEs. Data synthesis for mortality was not carried out since deaths were observed only in one study.

#### *CD4* + *T-cell count*

**Figure 5:**
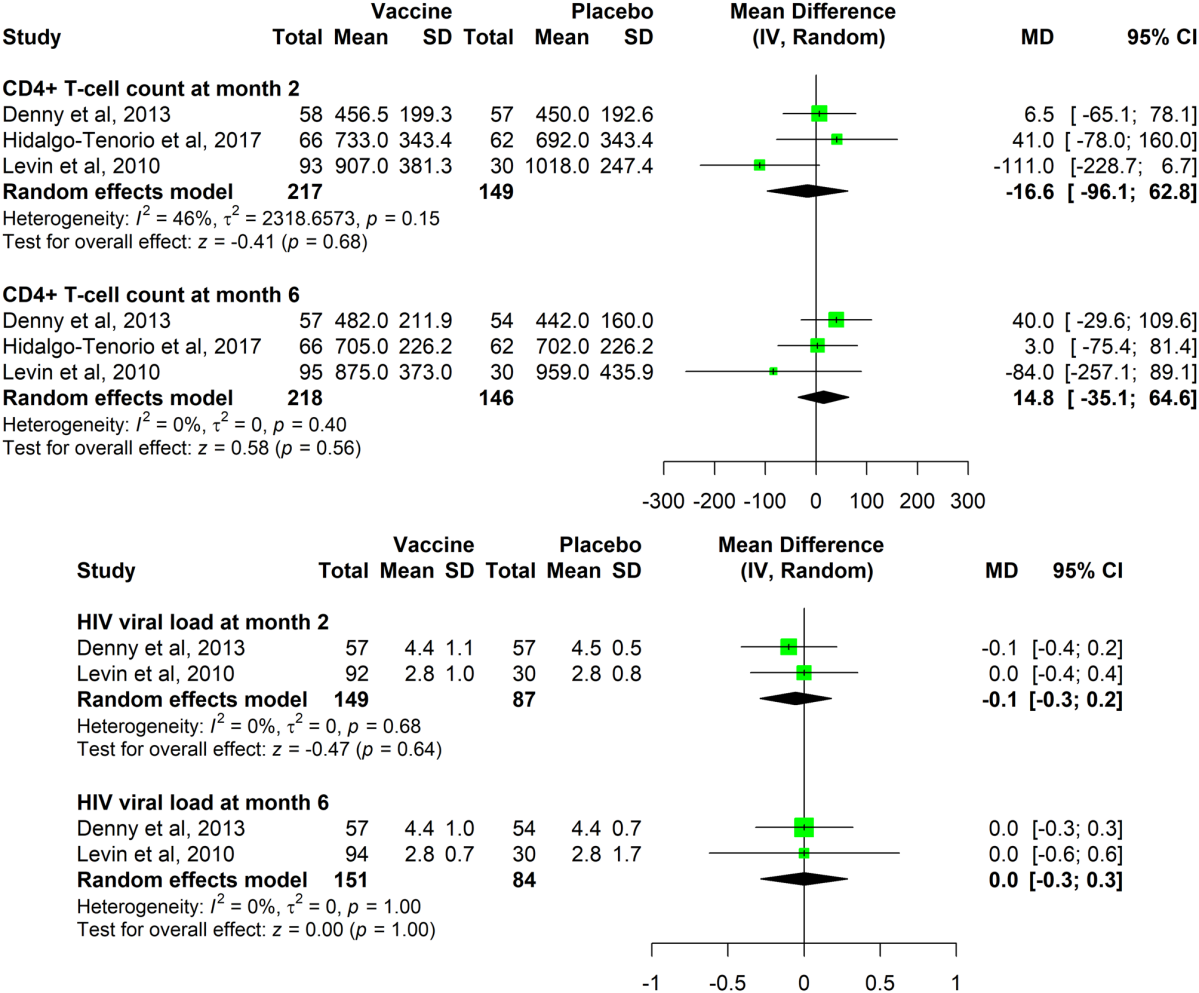
Data synthesis for CD4 cell count and HIV viral load outcomes.

The results concerning CD4 + T-cell count outcome (continuous outcome) are reported in Fig. 5. The combined MDs, considering equal variances between groups, were − 16.6 cells/µl (95% CI − 96.1 to 62.8) after the 2-nd vaccination and 14.8 cells/µl (95% CI − 35.1 to 64.6) after the end of the vaccination series.

#### HIV VL

The results concerning HIV VL outcome (continuous outcome) are reported in Fig. 5. The combined MDs, considering equal variances between groups, were − 0.1 log10 RNA copies/ml (95% CI − 0.3 to 0.2) after the 2-nd vaccination and 0.0 log_10_ RNA copies/ml (95% CI − 0.3 to 0.3) after the end of the vaccination series.

#### Completion rate

The results concerning the completion rate outcome are reported in Supplemental Fig. S3. The combined RR was 1.0 (95% CI 1.0–1.0).

## Subgroup analysis

No subgroup analysis was performed due to lack of the minimum number of studies.

### Summary of findings

As reported in Table 1, we rated all the relative effects as ‘high certainty’, since all the included studies were RCTs and we judged that there was no reason for downgrading the certainty of evidence. In particular, we found that the included studies were not affected by bias that could cause serious limitation to the results, as well as no imprecision of estimates or indirectness of evidence. We also assessed that none of our meta-analysis was affected by inconsistency. Moreover, publication bias was not a concern as, after searching on ClinicalTrials.gov registry, we did not find any completed studies before 2019 that have not yet been published.

**Table 1:** Summary of findings.

Outcomes^a^	Anticipated absolute effects^b^		Relative effect (95% CI)	No. of participants (studies)	Certainty of evidence (GRADE)	Comments
	Baseline risk in placebo group	Risk in vaccine group				
**Primary outcomes**						
Immunogenicity—HPV 16	Study population		MD = 4333.3 GMT EL.U/ml (2701.4–5965.1)	199 (2)	High ⊕ ⊕ ⊕ ⊕	
	16.6 GMT EL.U/ml	4350.0 GMT EL.U/ml				
Immunogenicity—HPV 18	Study population		MD = 1404.8 GMT EL.U/ml (414.8–2394.7)	200 (2)	High ⊕ ⊕ ⊕ ⊕	
	9.8 GMT EL.U/ml	1414.6 GMT EL.U/ml				
Not serious adverse events	Study population		RR = 1.0 (0.9–1.2)	375 (3)	High ⊕ ⊕ ⊕ ⊕	
	932 events per 1000 subjects	947 events per 1000 subjects				
Serious adverse events	Study population		RR = 0.6 (0.2–1.6)	949 (4)	High ⊕ ⊕ ⊕ ⊕	
	50 events per 1000 subjects	32 events per 1000 subjects				
**Secondary outcomes**						
CD4 + T-cell count—after vaccination series	Study population		MD = 14.8 cells/µl (− 35.1 to 64.6)	364 (3)	High ⊕ ⊕ ⊕ ⊕	
	702 cells/µl	717 cells/µl				
HIV viral load—after vaccination series	Study population		MD = 0.0 log_10_ RNA copies/ml (− 0.3 to 0.3)	235 (2)	High ⊕ ⊕ ⊕ ⊕	
	3.7 log_10_ RNA copies/ml	3.7 log_10_ RNA copies/ml				
Completion rate	Study population		RR = 1.0 (1.0–1.0)	949 (4)	High ⊕ ⊕ ⊕ ⊕	
	973 events per 1000 subjects	974 events per 1000 subjects				

Population: HIV-infected subjects.

Setting: any; studies conducted in: South Africa, Spain, USA, Brazil.

Intervention: bivalent or quadrivalent HPV Vaccine.

Comparison: placebo.

^a^The present summary of findings only considers primary and secondary outcome that were present in more than one study and that were eligible for data synthesis.

^b^For quantitative outcomes, the absolute baseline risk was the median value calculated over the mean values reported for the vaccine group in the included studies, whereas for dichotomous outcomes it was the median value calculated over the risks reported for the vaccine group in the included studies. The absolute risks in vaccine groups were calculated using the anticipated absolute effect in the placebo group and the relative effect measure. *95% CI* 95% confidence interval, *RR* risk ratio, *MD* mean difference. GRADE Working Group grades of evidence. High certainty: we are very confident that the true effect lies close to that of the estimate of the effect; Moderate certainty: we are moderately confident in the effect estimate: the true effect is likely to be close to the estimate of the effect, but there is a possibility that it is substantially different; Low certainty: our confidence in the effect estimate is limited: the true effect may be substantially different from the estimate of the effect; Very low certainty: we have very little confidence in the effect estimate: the true effect is likely to be substantially different from the estimate of effect. Criteria for grading of evidence: study design and risk of bias of included studies, indirectness of evidence, inconsistency and imprecision of relative effect estimates, publication bias.

## Discussion

### Summary of main results

The purpose of this systematic review is to summarize available data on the efficacy and safety of any types of HPV prophylactic vaccines in HIV + patients. Four RCTs conducted between 2008 and 2014 in Europe, America and Africa comprehending 950 participants living with HIV were included, 511 in the intervention group and 439 in the control group. Overall, we judged all the studies to be at moderate RoB.

As for the vaccine assessed, the immunogenicity and safety of the 4vHPV was tested in three trials, whereas only one evaluated the reactogenicity and safety of 2vHPV and none of the included studies reported the use of the 9vHPV.

There were no differences in the average CD4 + T-cell count and HIV VL, both after the 2-nd vaccination and after the end of the vaccination series.

Furthermore, no differences in the risk of AEs were observed.

Information on the remaining outcomes was scarce and that did not allow us to combine data.

### Overall completeness and applicability of evidence

EMBASE, PubMed and Cochrane Database of Systematic Reviewswere used to search for studies and data was extracted from the manuscripts and from the ClinicalTrials.gov registry.

The RCTs included were conducted among women, MSM and children, which represent the most vulnerable populations. However, HIV-infected men who have sex exclusively with women, injection drug users and transgender individuals were not considered as no RCTs including them were found.

Among the primary outcomes, HPV seroconversion and incidence of AEs were evaluated in all included studies, while other outcomes were analysed only in some of them: GMTs for all HPV vaccine types in two studies, persistent anal and oral HPV infection of any HPV type in one study. No data were available on the efficacy of vaccine against CIN of grades 2 or 3. Regarding secondary outcomes, HIV VL and CD4 + T-cell count were reported in two studies.

As yet, no studies on second-generation 9vHPV in HIV patients have been published, although it is assumed that the 9vHPV will elicit an immune response. Clinical trials are currently underway to provide more extensive data on the efficacy and safety of the 9vHPV vaccine in the prevention of HPV disease in individuals with HIV^29^.

In Spain, a clinical trial^30^ is assessing immunogenicity and safety of 9vHPV vaccine among 158 adult HIV + women. Another RCT^31^ in the USA is assessing the immunogenicity of the 9vHPV vaccine in MSM infected by HIV. Furthermore, the COVENANT trial^32^ is evaluating the effect of HPV vaccine in reducing lesions in HIV + women with high-grade cervical lesions.

### Certainty of the evidence

For all the outcomes that were analysed, we found a high degree of certainty of the evidence of the efficacy and safety of HPV vaccine in HIV + patients.

### Potential biases in the review process

With regards to AEs, there was high heterogeneity in their definitions, nor were the time-points of assessment equal across studies. This latter issue was also present for other outcomes, as reported in Supplemental Table S3. In this review, we focused on the overall frequency of not serious and serious AEs, as well as the all-causes mortality rates.

We are aware of some main limitations that could affect the results of this review. Firstly, the populations considered in the studies were heterogeneous. Secondly, the clinical outcomes of the HPV vaccination, such as infections and high grade lesions or neoplasms, were assessed in only one study and the long term outcomes were not assessed in any RCT. As minor limitations, the time-points used were decided after seeing the time-points reported in the primary studies and small approximations on summary measures of some outcomes, as described in the previous sections, were made.

### Context for this review

The development of 2vHPV, 4vHPV e 9vHPV vaccines against HPV represents an opportunity to control and possibly eradicate the cancers associated to infection.

People HIV-infected are at increased risk of acquisition of HPV infection leading on to the development of cervical cancer and other genital illness^33^. In addition to high incidence, multiple HPV infections are more common among HIV + patients; a higher risk of multiple HPV types was found among HIV + MSM as compared to HIV- MSM^34^. In comparison to HIV- subjects, they have an increased HPV persistence, higher risk of HPV-related tumours and faster disease progression. Thus, HPV prevention is very important in HIV + patients^35^. Currently HPV vaccination is recommended for use among adult females and males up to 26 years, as well as MSM and subjects with immunocompromising conditions including those with HIV infection^36,37^.

### Agreements and disagreements with other systematic reviews

The World Health Organization (WHO) and Centers for Disease Control and Prevention (CDC) recommend HPV vaccination with a three-dose schedule (at 0, 1–2, and 6 months) for all HIV + up to 26 years of age if they have not already been vaccinated^36,38^. The availability of limited information about the efficacy of HPV vaccines in people living with HIV has recently been highlighted both by WHO and in a systematic review^36,39^.

Two Cochrane systematic reviews have been conducted to assess the efficacy of HPV vaccines. However, Arbyn et al.^40^ did not consider HIV + patients, while Bergman et al.^39^ included both HIV- and HIV + subjects, but they did not consider some outcomes of our interest. In particular, HIV VL and CD4 + T-cell count were not analysed in Bergman et al.^39^.

The results of this systematic review agreed with those obtained by Bergman et al. on common outcomes^39^. We both observed that vaccines are effective and safe but there is still limited evidence in people living with HIV. The authors suggested that further studies on the duration of protection and on the effect of declining immunity are needed.

A systematic review and meta-analysis was recently published on the safety and efficacy of vaccination for people living with HIV that considered 24 studies, which included RCTs and non-RCT. In this review, studies reporting the difference between HIV + and HIV- people after HPV vaccination were also considered^41^. As for the seroconversion rates, the antibodies levels and occurrence of AEs, the results of our study agree with those of Zhan et al.^41^. An important difference concerns instead the level of CD4 + T-cell count in the vaccine group which in our analysis does not show significant differences when compared with placebo group.

### Implications for practice

Few RCTs on HIV + patients comparing efficacy and safety of HPV vaccination against placebo were carried out. Their results suggested that after the HPV vaccination, seroconversion rates were nearly 100% in women, men and children and a high antibody titers was observed for all HPV genotypes included in the vaccines. Vaccination in patients with HIV infection is safe as the number of serious AEs is similar between HIV + subjects treated with vaccines and placebo. Moreover, vaccines did not cause variations in CD4 + T-cell count and HIV VL. There is evidence on the benefit of HPV vaccine in HIV + patients, although efficacy on prevention of infections and HPV-related neoplasia still remains unknown. The results of our meta-analyses, despite being based on a few studies, suggest that HPV vaccine can be safely used in HIV + patients. Evidence on the efficacy and safety profile is available for 2vHPV, and 4vHPV vaccines. Regarding 9vHPV vaccine, no RCT in HIV + individuals is currently available. The compliance of the vaccination for the HIV + population represents a relevant issue that can be addressed by providing direct access to vaccination programs in hospital departments were HIV patients are cared for and by implementation effective communication campaigns on the importance of primary prevention.

### Implications for research

As AEs were reported in all RCTs in a heterogeneous manner, we believe that there is a need to standardise the way the information is collected and reported.

Another important aspect which is worth considering for future research concerns the lack of information on some important outcomes. For example, the prevention of anogenital warts was only addressed in one study, whereas no study considered cervical intraepithelial neoplasia and cancer as outcomes.

Further studies are needed to assess the effectiveness of the HPV vaccines in preventing infections and neoplasms in people living with HIV.

## Supplementary information

Supplementary Figure Legend

Supplementary FigureS1

Supplementary FigureS2

Supplementary Figure S3

Supplementary Table S1

Supplementary Table S2

SupplementaryTable S3

SupplementaryTable S4
