# Supplementary material for: Efficacy and safety of human papillomavirus vaccination in HIV-infected patients: a systematic review and meta-analysis
Source: Sci Rep. 2021 Mar 2;11:4954. doi: 10.1038/s41598-021-83727-7 (PMC7925667; doi:10.1038/s41598-021-83727-7)
Supplement: Supplementary file 1 — Supplementary Figure Legend [file 41598_2021_83727_MOESM1_ESM.docx]

**Efficacy and safety of human papillomavirus vaccination in HIV-infected patients: a systematic review and meta-analysis**

Antonella Zizza^1,+^, Federico Banchelli^2,+^, Marcello Guido^3,4,+,*^, Claudia Marotta^5,6^, Francesco Di Gennaro^7^, Walter Mazzucco^5,8,9^, Vanna Pistotti^10^, Roberto D’Amico^2^

^1^National Research Council, Institute of Clinical Physiology, Lecce, 73100, Italy

^2^University of Modena and Reggio Emilia, Department of Medical and Surgical Sciences, Modena, 41100, Italy

^3^University of Salento, Laboratory of Hygiene, Department of Biological and Environmental Sciences and Technologies, Lecce, 73100, Italy

^4^University of Genoa, Inter-University Centre of Research on Influenza and other Transmissible Infections (CIRI-IT), Genoa, 16100, Italy

^5^University of Palermo, Department of Health Promotion, Maternal and Infant Care, Internal Medicine and Medical Specialties (PROMISE), Palermo, 90100, Italy

^6^IRCCS Neuromed, Medical Direction, Pozzilli (IS), 86170, Italy

^7^University of Bari “Aldo Moro”, Department of Infectious Diseases, Bari, 70124, Italy

^8^COVID-19 Sicilian regional reference Lab, Palermo University Hospital (AOUP) “P. Giaccone”, Clinical Epidemiology and Cancer Registry Unit, Palermo, 90100, Italy

^9^Cincinnati College of Medicine, Department of Pediatric - Division of Biostatistics and Epidemiology, Cincinnati Children’s Hospital Medical Center, Cincinnati (OH), USA

^10^Independent researcher

*****[marcello.guido@unisalento.it](mailto:marcello.guido@unisalento.it)

^+^these authors contributed equally to this work

******Corresponding author*:

Marcello Guido, Ph.D.

Laboratory of Hygiene, Department of Biological and Environmental Sciences and Technologies

University of Salento

Via Prov.le Lecce-Monteroni, 165, Lecce, 73100, Italy

Phone: +39-832-298686

Fax: +39-832-298626

email: [marcello.guido@unisalento.it](mailto:marcello.guido@unisalento.it)

**Supplementary figure legend:**

**Supplementary Figure 1.** Data synthesis for safety outcomes.

**Supplementary Figure 2.** Data synthesis for anal or oral infection and anal neoplasia outcomes.

**Supplementary Figure 3.** Data synthesis for completion rate outcome.
