# Supplementary figures and images for: Efficacy and safety of human papillomavirus vaccination in HIV-infected patients: a systematic review and meta-analysis
Source: Sci Rep. 2021 Mar 2;11:4954. doi: 10.1038/s41598-021-83727-7 (PMC7925667; doi:10.1038/s41598-021-83727-7)

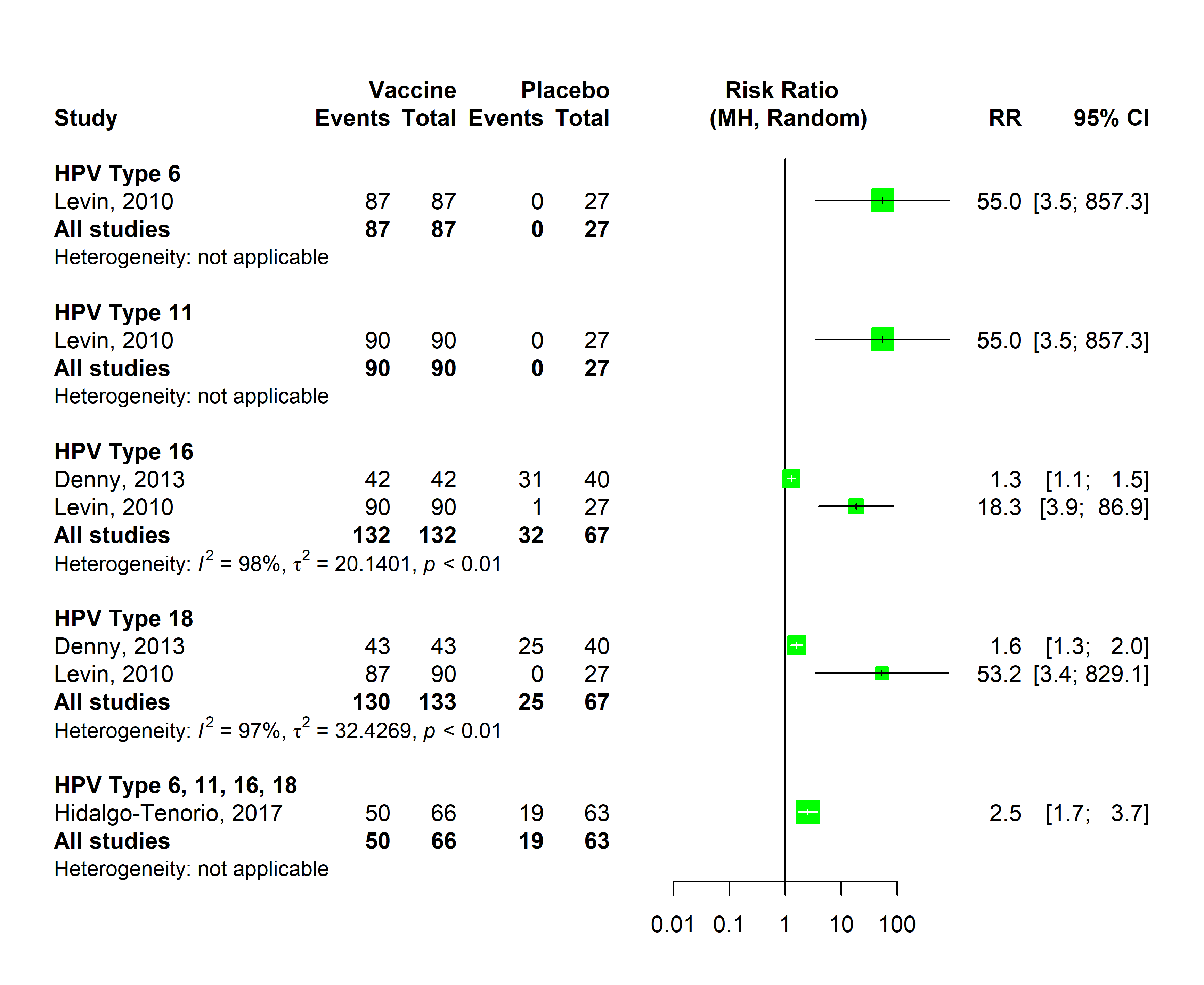

Supplement: Supplementary file 2 — Supplementary FigureS1 [file 41598_2021_83727_MOESM2_ESM.tiff]

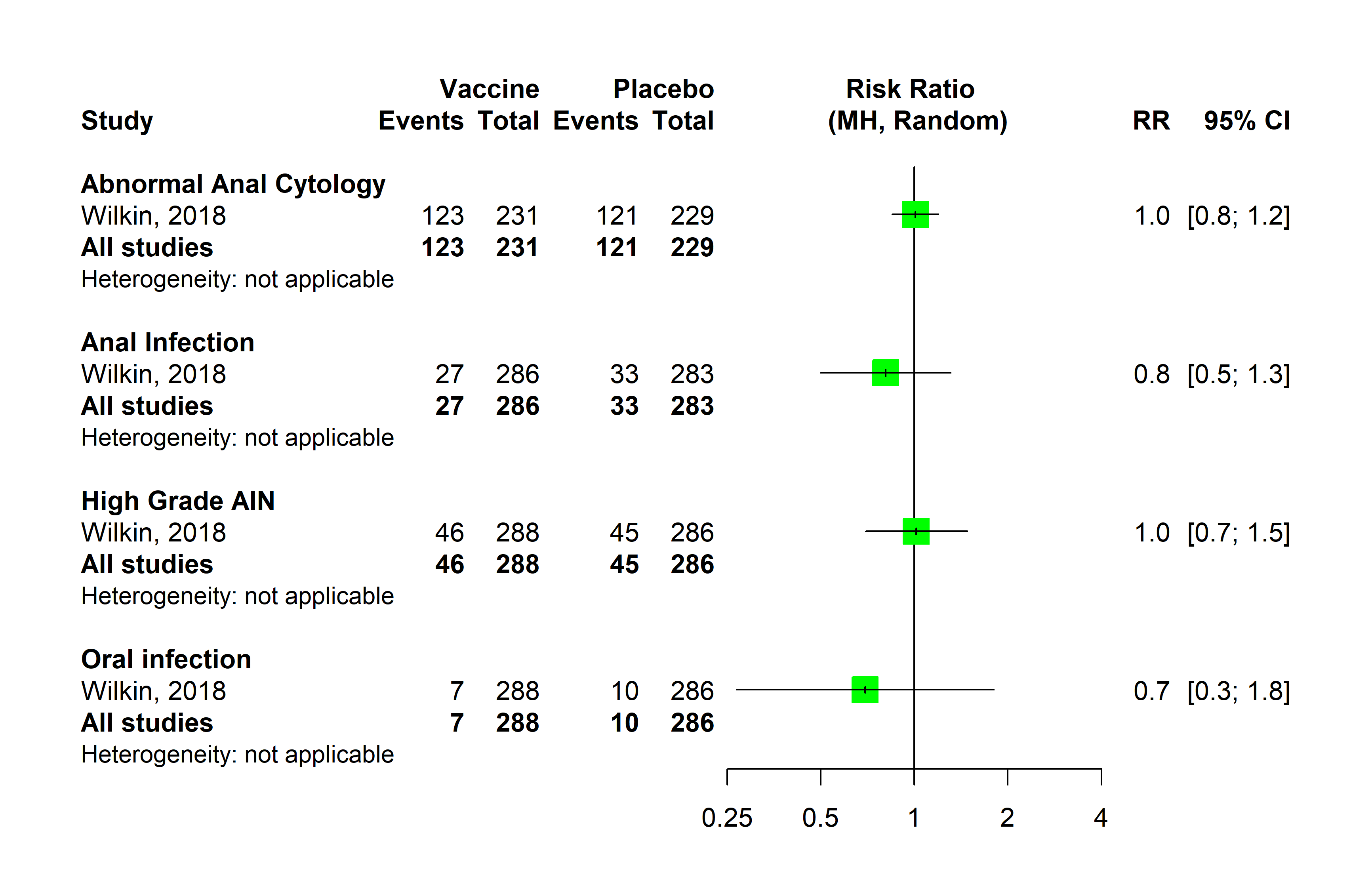

Supplement: Supplementary file 3 — Supplementary FigureS2 [file 41598_2021_83727_MOESM3_ESM.tiff]

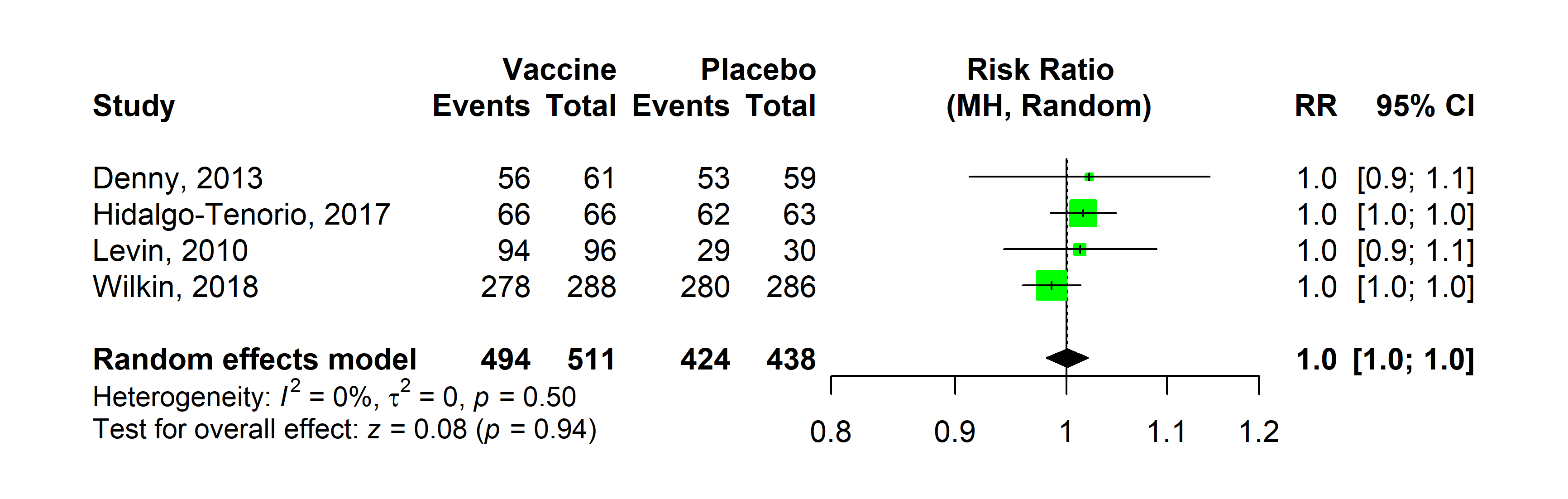

Supplement: Supplementary file 4 — Supplementary Figure S3 [file 41598_2021_83727_MOESM4_ESM.tiff]
