# Supplementary material for: Efficacy and safety of human papillomavirus vaccination in HIV-infected patients: a systematic review and meta-analysis
Source: Sci Rep. 2021 Mar 2;11:4954. doi: 10.1038/s41598-021-83727-7 (PMC7925667; doi:10.1038/s41598-021-83727-7)
Supplement: Supplementary file 5 — Supplementary Table S1 [file 41598_2021_83727_MOESM5_ESM.docx]

**Supplementary table 1.** Search strategy used for this review.

| **N** | **Query** | **Results** |
| --- | --- | --- |
| **PubMed** | | |
| #11 | Search (((((#6) AND random*)) OR (((((#1 OR #2 OR #3))) AND ((HIV Infections[MeSH] OR HIV[MeSH] OR hiv OR” hiv 1”OR “hiv 2”OR hiv1 OR hiv2 OR” hiv infect*”OR “human immunodeficiency virus” OR “human immunedeficiency virus”OR “human immune deficiency virus”))) AND Randomized Controlled Trial[ptyp]))) AND 2018:2019 [edat]. | [3](https://www.ncbi.nlm.nih.gov/pubmed/?cmd=HistorySearch&querykey=52) |
| #10 | Search 2018:2019 [edat]. | 1645216 |
| #9 | Search (((#6) AND random*)) OR (((((#41 OR #42 OR #43))) AND ((HIV Infections[MeSH] OR HIV[MeSH] OR hiv OR” hiv 1”OR “hiv 2”OR hiv1 OR hiv2 OR” hiv infect*”OR “human immunodeficiency virus” OR “human immunedeficiency virus”OR “human immune deficiency virus”))) AND Randomized Controlled Trial[ptyp]) | [59](https://www.ncbi.nlm.nih.gov/pubmed/?cmd=HistorySearch&querykey=49) |
| [#8](https://www.ncbi.nlm.nih.gov/pubmed/advanced) | Search (#6) AND random* | [59](https://www.ncbi.nlm.nih.gov/pubmed/?cmd=HistorySearch&querykey=48) |
| [#7](https://www.ncbi.nlm.nih.gov/pubmed/advanced) | Search (((#1 OR #2 OR #3))) AND ((HIV Infections[MeSH] OR HIV[MeSH] OR hiv OR” hiv 1”OR “hiv 2”OR hiv1 OR hiv2 OR” hiv infect*”OR “human immunodeficiency virus” OR “human immunedeficiency virus”OR “human immune deficiency virus”)) Filters: Randomized Controlled Trial | [24](https://www.ncbi.nlm.nih.gov/pubmed/?cmd=HistorySearch&querykey=47) |
| [#6](https://www.ncbi.nlm.nih.gov/pubmed/advanced) | Search (((#1 OR #2 OR #3))) AND ((HIV Infections[MeSH] OR HIV[MeSH] OR hiv OR” hiv 1”OR “hiv 2”OR hiv1 OR hiv2 OR” hiv infect*”OR “human immunodeficiency virus” OR “human immunedeficiency virus”OR “human immune deficiency virus”)) | [1108](https://www.ncbi.nlm.nih.gov/pubmed/?cmd=HistorySearch&querykey=46) |
| [#5](https://www.ncbi.nlm.nih.gov/pubmed/advanced) | Search (HIV Infections[MeSH] OR HIV[MeSH] OR hiv OR” hiv 1”OR “hiv 2”OR hiv1 OR hiv2 OR” hiv infect*”OR “human immunodeficiency virus” OR “human immunedeficiency virus”OR “human immune deficiency virus”) | [395674](https://www.ncbi.nlm.nih.gov/pubmed/?cmd=HistorySearch&querykey=45) |
| [#4](https://www.ncbi.nlm.nih.gov/pubmed/advanced) | Search (#1 OR #2 OR #3) | [19861](https://www.ncbi.nlm.nih.gov/pubmed/?cmd=HistorySearch&querykey=44) |
| [#3](https://www.ncbi.nlm.nih.gov/pubmed/advanced) | Search ((“human papillomavirus vaccin*” OR “human papilloma virus vaccin*” OR “ HPV vaccin*” OR “HPV L1 vaccin*” OR ” human papillomavirus L1 vaccin*” OR “alphapapillomavirus vaccin*” OR Gardasil OR “Merck HPV vaccin*” OR “Merck HPV vaccin*” OR” Merck human papillomavirus vaccin*” OR “Merck human papillomavirus vaccin*” OR Cervarix OR “GSK HPV vaccin*” OR “GSK HPV vaccin*” OR “GSK human papillomavirus vaccin*” OR “GSK human papillomavirus vaccin*” OR “GlaxoSmithKline HPV vaccin*” OR “GlaxoSmithKline HPV vaccin*” OR “GlaxoSmithKline human papillomavirus vaccin*” OR “GlaxoSmithKline human papillomavirus vaccin*” OR “quadrivalent HPV vaccin*” OR “quadrivalent HPV vaccin*” OR QHPV OR HPV4 OR ”bivalent HPV vaccin*” OR “bivalent HPV vaccin*” OR BHPV OR HPV2 OR “human papillomavirus type 6 vaccin*” OR “HPV type 6 vaccin*” OR “HPV6 vaccin*” OR “human papillomavirus type 11 vaccin*” OR “HPV type 11 vaccin*” OR “HPV11 vaccin*” OR ” human papillomavirus type 16 vaccin*” OR “HPV type 16 vaccin*” OR “HPV16 vaccin*” OR “human “papillomavirus type 18 vaccin*” OR “HPV type 18 vaccin*” OR “HPV18 vaccin*”)) OR (Papillomavirus Vaccine[MeSH] OR Alphapapillomavirus[MeSH]) | [13849](https://www.ncbi.nlm.nih.gov/pubmed/?cmd=HistorySearch&querykey=43) |
| [#2](https://www.ncbi.nlm.nih.gov/pubmed/advanced) | Search “nonavalent HPV vaccin*” OR “nine valent” OR ninevalent | [145](https://www.ncbi.nlm.nih.gov/pubmed/?cmd=HistorySearch&querykey=42) |
| [#1](https://www.ncbi.nlm.nih.gov/pubmed/advanced) | Search "HPV 6" OR "HPV 11" OR "HPV 16" OR "HPV 18" OR "HPV 31" OR "HPV 33" OR "HPV 45" OR "HPV 52" OR "HPV 58 " | [8909](https://www.ncbi.nlm.nih.gov/pubmed/?cmd=HistorySearch&querykey=41) |
| **Embase** | | |
| #18 | #16 AND [randomized controlled trial]/lim NOT ('systematic review*' OR review*) AND [embase]/lim AND [1-1-2018]/sd | 4 |
| #17 | #16 AND [randomized controlled trial]/lim NOT ('systematic review*' OR review*) AND [embase]/lim | 24 |
| #16 | #14 AND #15 | 2271 |
| #15 | 'human immunodeficiency virus infection'/exp OR hiv:ti,ab OR 'hiv 1':ti,ab OR 'hiv 2':ti,ab OR hiv1:ti,ab OR hiv2:ti,ab OR 'hiv infect*':ti,ab OR 'human immunodeficiency virus':ti,ab OR 'human immunedeficiency virus':ti,ab OR 'human immune deficiency virus':ti,ab | 504314 |
| #14 | #1 OR #3 OR #4 OR #5 OR #6 OR #7 OR #8 OR #9 OR #10 OR #11 OR #12 OR #13 | 31033 |
| #13 | 'human papillomavirus vaccin*' OR 'human papilloma virus vaccin*' OR 'hpv vaccin*' OR 'hpv l1 vaccin*' OR 'human papillomavirus l1 vaccin*' OR 'alphapapillomavirus vaccin*' OR gardasil | 11552 |
| #12 | 'merck hpv vaccin*' OR cervarix OR 'gsk hpv vaccin*' | 1780 |
| #11 | 'glaxosmithkline hpv vaccin*' | 0 |
| #10 | 'gsk human papillomavirus vaccin*' | 0 |
| #9 | 'glaxosmithkline human papillomavirus vaccin*' OR 'quadrivalent hpv vaccin*' | 557 |
| #8 | qhpv OR hpv4 OR 'bivalent hpv vaccin*' | 385 |
| #7 | bhpv OR hpv2 OR 'human papillomavirus type 6 vaccin*' OR 'hpv type 6 vaccin*' OR 'hpv6 vaccin*' | 111 |
| #6 | 'human papillomavirus type 11 vaccin*' OR 'hpv type 11 vaccin*' OR 'hpv11 vaccin' | 3 |
| #5 | 'human papillomavirus type 16 vaccin*' | 14 |
| #4 | 'hpv type 16 vaccin*' OR 'hpv16 vaccin*' OR 'human papillomavirus type 18 vaccin*' | 34 |
| #3 | 'wart virus vaccine'/exp OR 'alphapapillomavirus'/exp OR 'alphapapillomavirus' | 23882 |
| #2 | 'nonavalent hpv vaccin*' OR 'nine valent' OR ninevalent | 140 |
| #1 | 'hpv 6' OR 'hpv 11' OR 'hpv 16' OR 'hpv 18' OR 'hpv 31' OR 'hpv 33' OR 'hpv 45' OR 'hpv 52' OR 'hpv 58' | 11028 |
| **Cochrane Library** | | |
| #17 | #16 and #12 with Publication Year from 2018 to 2019, in Trials | 1 |
| #16 | #4 or #15 | 24615 |
| #15 | hiv OR ”hiv 1” OR “hiv 2” OR hiv1 OR hiv2 OR ”hiv infect*” | 24615 |
| #14 | “human mmunodeficiency virus” OR “human immunedeficiency virus” OR “human immune deficiency virus” | 104 |
| #13 | #12 or #4 or #7 or #9 | 575 |
| #12 | #1 or #2 or #3 | 535 |
| #11 | "HPV 18 Vaccin*" | 0 |
| #10 | “HPV type 18 vaccin*” | 0 |
| #9 | “HPV16 vaccin*” OR “human papillomavirus type 18 vaccin*” | 1 |
| #8 | “human papillomavirus type 11 vaccin*” OR “HPV type 11 vaccin*” OR “HPV11 vaccin*” OR ” human papillomavirus type 16 vaccin*” OR “HPV type 16 vaccin*” | 0 |
| #7 | “quadrivalent HPV vaccin*” OR QHPV OR HPV4 OR ”bivalent HPV vaccin*” OR “bivalent HPV vaccin*” OR BHPV OR HPV2 OR “human papillomavirus type 6 vaccin*” OR “HPV type 6 vaccin*” OR “HPV6 vaccin*” | 73 |
| #6 | “GSK human papillomavirus vaccin*” OR “GSK human papillomavirus vaccin*” OR “GlaxoSmithKline HPV vaccin*” OR “GlaxoSmithKline HPV vaccin*” OR “GlaxoSmithKline human papillomavirus vaccin*” OR “GlaxoSmithKline human papillomavirus vaccin*” OR “quadrivalent HPV vaccin*” | 0 |
| #5 | Gardasil OR “Merck HPV vaccin*” OR “Merck HPV vaccin*” OR” Merck human papillomavirus vaccin*” OR “Merck human papillomavirus vaccin*” OR Cervarix OR “GSK HPV vaccin*” OR “GSK HPV vaccin*” | 0 |
| #4 | “HPV vaccin*” OR “HPV L1 vaccin*” OR ” human papillomavirus L1 vaccin*” OR “alphapapillomavirus vaccin*” | 3 |
| #3 | “human papillomavirus vaccin*” OR “human papilloma virus vaccin*” | 3 |
| #2 | “nonavalent HPV vaccin*” OR “nine valent” OR ninevalent | 18 |
| #1 | "HPV 6" OR "HPV 11" OR "HPV 16" OR "HPV 18" OR "HPV 31" OR "HPV 33" OR "HPV 45" OR "HPV 52" OR "HPV 58 " | 519 |
