# Supplementary material for: Efficacy and safety of human papillomavirus vaccination in HIV-infected patients: a systematic review and meta-analysis
Source: Sci Rep. 2021 Mar 2;11:4954. doi: 10.1038/s41598-021-83727-7 (PMC7925667; doi:10.1038/s41598-021-83727-7)
Supplement: Supplementary file 6 — Supplementary Table S2 [file 41598_2021_83727_MOESM6_ESM.docx]

**Supplementary table 2.** Characteristics of the included studies.

| **Denny, 2013** | |
| --- | --- |
| Methods | **Phase I/II, partially blind (Double blind) and randomized placebo-controlled trial** |
| Participants | **Country, South Africa**  120 women 18-25 years with HIV infection [mean ± SD: HIV+/HPV (n=61), 21.6 ± 2.21; HIV+/ALU (n=59), 22.7 ± 1.7].  **Inclusion criteria:** Women aged 18-25 years with an intact cervix who reported no more than six lifetime sexual partners. Sexually active women had to have a normal colposcopy and a normal cervical cytology or no greater than atypical squamous cells of undetermined significance at the screening visit. All women had to be willing to undergo HIV counselling and testing and to be informed of their HIV status. HIV-positive women had to be in WHO Clinical Stage 1. Women on ART had to be compliant with treatment and have a HIV viral load ≤ 400 copies/mm^3^ for at least 6 months.  **Exclusion criteria:** Pregnant women were excluded. |
| Interventions | HIV-positive women were randomized (1:1) to receive the HPV-16/18 AS04-adjuvanted vaccine (Cervarix®*, GlaxoSmithKline Vaccines) (HIV+/HPV group) or Al(OH)_3_ control (HIV+/ALU group) at 0, 1, and 6 months in a double-blind manner using a centralized internet-based randomization system. |
| Outcomes | Seroconversion, immunogenicity and safety: Seroconversion rates for each HPV vaccine types and total for HIV+/HPV group; GMTs for each HPV vaccine types in vaccine and control group; Incidence (n, %) of AEs (overall). |
| Notes | Solicited AEs were recorded by a trained field worker daily for 7 days after each vaccination. Severity of solicited AEs was graded on a scale of 0 (absent) to 3 (preventing normal activities). Unsolicited AEs were recorded for 30 days after each vaccination. SAEs, medically significant adverse events, new-onset chronic diseases (NOCDs), pregnancies and their outcome were recorded up to month 12. Clinically relevant abnormalities in hematological and biochemical parameters were monitored. |
| **Hidalgo-Tenorio, 2017** | |
| Methods | **Randomized, double blind, placebo‐controlled trial** |
| Participants | **Country, Spain**  129 men who have sex with man (MSM) HIV positive [mean ± SD: HIV+/HPV (n=66), 37.3 ± 10.6; HIV+/placebo (n=63), 40.5 ± 10.02].  **Inclusion criteria:** HIV-positive MSM patients of ≥18 years of age who, at the time of study inclusion were not infected simultaneously by the four genotypes of HPV that the quadrivalent vaccine addresses; Patients who had a normal high-resolution anoscopy (HRA) at screening for inclusion or only had condylomas and/or low squamous intraepithelial lesion (LSIL) in anal biopsy.  **Exclusion criteria:** HIV MSM patients who had simultaneous anal infection with the four genotypes addressed by the vaccine, and who at least had HPV genotypes 16 and 18; Active opportunist infection at the time of recruitment into the study; Patients who, in screening anoscopy had HSIL, or ASCC or had received treatment for these lesions; History of allergy to aluminum and/or yeast extract excipient. |
| Interventions | Quadrivalent (HPVs 6/11/16/18) vaccine (Gardasil©; Merck Research Laboratories) and placebo: subjects were randomly assigned in a 1:1 ratio to receive vaccine or placebo at day 1, month 2 and month 6. |
| Outcomes | Seroconversion and safety: Seroconversion rates for total HPV vaccine types for HIV-MSM vaccine and placebo; Incidence (n, %) of AEs. |
| Notes | The AEs assessment system employed was a questionnaire that included the most frequent local reactions such as fever, nausea, vomiting, dizziness, syncope, headache and others such as allergic reaction, pruritus, difficulty breathing and/or wheezing. Rare occurrences included lymphadenopathies, chest and lower-limb pain, confusion, chills, muscle pain. The AEs were graded on a scale of 1–4. In case of AE grade 4, the blind of the vial administered was broken and, if the code identified the vaccine, the reaction was communicated immediately to the relevant drug-vigilance authorities. |
| **Levin, 2010** | |
| Methods | **Phase II, randomised clinical trial** |
| Participants | **Country, USA**  **Participants: 126** Children (girls and boys) with HIV infection HIV positive [mean; CI95%: HIV+/HPV (n=96), 10 (9.7-10.3); HIV+/placebo (n=30), 9.9 (9.4-10-4)].  **Inclusion criteria:** Children >7 to <12 years with HIV infection if their baseline CD4% was ≥15. At least 3 months of highly active antiretroviral therapy (HAART) was required for subjects with a CD4% <25.  **Exclusion criteria: O**ther immunosuppressive diseases or medications; Other significant acute or chronic illness; Other vaccinations within 2–3 weeks (depending on vaccine type) before or after study vaccine; Significant abnormalities in hematologic or chemistry tests; Receipt of blood-derived products within 6 months before or during the study. |
| Interventions | Intervention: Quadrivalent (HPVs 6/11/16/18) recombinant vaccine (Gardasil), administered by intramuscular injection at 0, month 1 and 7. Comparison: identical placebo, 0.5 mL, administered by intramuscular injection. |
| Outcomes | Seroconversion, immunogenicity and safety:   - Seroconversion rates for each HPV vaccine types for HIV+/HPV and HIV+/placebo; - GMTs for each HPV vaccine types in vaccine and control group; - Incidence (%) of AEs occurred within 14 days of each vaccination. |
| Notes | **AEs.** Subjects were observed in clinic for 30 minutes’ post vaccination. A report card of relevant signs and symptoms was maintained by the caregiver for 15 days after each injection. Body temperature was recorded for 5 days beginning after the injection. Telephone contact with the caregiver was made on the third day after each injection to inquire about reactions. The caregiver was instructed to immediately report unusual injection site reactions. A clinic visit was required within 24 hours whenever the study coordinator considered that a reaction might be ≥grade 3 (Division of AIDS Table for Grading the Severity of AEs: http://rcc.tech-res.com/safetyandpharmacyvigilance). |
| **Wilkin, 2018** | |
| Methods | **Phase III, randomized, double-blind, placebo-controlled trial** |
| Participants | **Country,** USA and Brazil **(**24 sites)  **Participants 575** HIV-infected MSM and women ≥27 years [median; IQR; HIV+/HPV (n=288), 47 (40,52); HIV+/placebo (n=287), 48 (42,53)].  **Inclusion criteria:** HIV-1 infection; Age ≥27years; Safety laboratory tests; For men receptive penile-anal sex or oral-anal sex with another man within one year; Ability to provide informed consent. 30% of male participants and 50% of female participants were required to have HSIL on histologic analysis of anal biopsies (bHSIL).  **Exclusion criteria:** History of HPV-related cancer; Anal HSIL or condyloma treatment within 6 months; Prior HPV vaccination; Anticoagulant use; Allergy to vaccine components; Active drug or alcohol use; Other condition that would interfere with study requirements, bleeding diatheses, systemic antineoplastic or immunomodulatory treatment, pregnancy or breastfeeding. |
| Interventions | HPV quadrivalent (Types 6, 11, 16 and 18) vaccine (Merck and Co. Inc., Kenilworth, NJ) at 0, 8, 24 weeks. The placebo for male participants was the vaccine adjuvant with no viral-like particles; female participants received 0.9% saline as placebo was no longer available from Merck. |
| Outcomes | Seroconversion, immunogenicity and safety: Seroconversion rates for total HPV vaccine types for HIV+ vaccine group and HIV+ control group; Grade 3 or 4 AEs related to vaccination; Time to first new persistent infection of any qHPV type, defined as qHPV-type infection confirmed by PCR at two consecutive 6-month assessments; Persistent anal and oral HPV infection, detection of bHSIL at week 52 or later, anal cytological outcomes |
| Notes | AEs were solicited during clinical assessments and graded using Division of AIDS Table for Grading the Severity of Adult AEs Version 1.0, December 2004 (available at http://rsc.tech res.com/safetyandpharmacovigilance). Seroconversion was not defined as an outcome in the Methods section, however the author reported it in the Results section. |
