# Supplementary material for: Efficacy and safety of human papillomavirus vaccination in HIV-infected patients: a systematic review and meta-analysis
Source: Sci Rep. 2021 Mar 2;11:4954. doi: 10.1038/s41598-021-83727-7 (PMC7925667; doi:10.1038/s41598-021-83727-7)
Supplement: Supplementary file 7 — SupplementaryTable S3 [file 41598_2021_83727_MOESM7_ESM.docx]

**Supplementary table 3.** Summary of time points at which outcomes were reported, for each outcome in each included study.

|  |  | Month 0 | Month 1 | Month 2 / Week 8 | Month 3 / week 12 | Month 4 | Month 6 / Week 28 | Month 7 | Month 10 | Month 11 / Week 48 | Month 12  / Week 52 |
| --- | --- | --- | --- | --- | --- | --- | --- | --- | --- | --- | --- |
| Seroconversion  (dichotomous outcome) | Denny, 2013 |  |  | X |  |  |  | X |  |  | X |
|  | Hidalgo-Tenorio, 2017 |  |  |  |  |  |  | X |  |  |  |
|  | Levin, 2010 |  |  |  |  |  | X |  |  |  |  |
|  | Wilkin, 2018 |  |  |  |  |  |  |  |  |  |  |
| Seroconversion  (continuous outcome) | Denny, 2013 |  |  | X |  |  |  | X |  |  | X |
|  | Hidalgo-Tenorio, 2017 |  |  |  |  |  |  |  |  |  |  |
|  | Levin, 2010 |  |  |  |  |  | X |  |  |  |  |
|  | Wilkin, 2018 |  |  |  |  |  |  |  |  |  |  |
| Abnormal Anal Citology | Denny, 2013 |  |  |  |  |  |  |  |  |  |  |
|  | Hidalgo-Tenorio, 2017 |  |  |  |  |  |  |  |  |  |  |
|  | Levin, 2010 |  |  |  |  |  |  |  |  |  |  |
|  | Wilkin, 2018 |  |  |  |  |  |  |  |  |  | X |
| Adverse events | Denny, 2013 |  |  |  |  |  |  | X |  |  | X |
|  | Hidalgo-Tenorio, 2017 | X |  | X |  |  | X |  |  |  |  |
|  | Levin, 2010 |  |  |  |  |  | X |  |  |  |  |
|  | Wilkin, 2018 |  |  |  |  |  |  |  |  | X^(a)^ |  |
| Mortality | Denny, 2013 |  |  |  |  |  |  |  |  |  | X |
|  | Hidalgo-Tenorio, 2017 | X |  | X |  |  | X |  |  |  |  |
|  | Levin, 2010 |  |  |  |  |  |  |  |  |  |  |
|  | Wilkin, 2018 |  |  |  |  |  |  |  |  | X |  |
| CD4 cell count | Denny, 2013 |  | X | X |  | X | X | X | X |  | X |
|  | Hidalgo-Tenorio, 2017 | X |  | X |  |  | X |  |  |  |  |
|  | Levin, 2010 |  |  | X | X |  | X^(b)^ |  |  |  |  |
|  | Wilkin, 2018 |  |  |  |  |  |  |  |  |  |  |
| HIV viral load | Denny, 2013 |  | X | X |  | X | X | X | X |  | X |
|  | Hidalgo-Tenorio, 2017 |  |  |  |  |  |  |  |  |  |  |
|  | Levin, 2010 |  |  | X | X |  | X^(b)^ |  |  |  |  |
|  | Wilkin, 2018 |  |  |  |  |  |  |  |  |  |  |

**Notes:** An “X” sign represents the presence of the appropriate summary measures concerning the outcome and the study as indicated by the row labels, at the time point as indicated by the column label, for both vaccine and placebo groups; time points in Levin and Wilkin were reported as weeks and were rescaled to months by dividing them per the average number of weeks in a month in a not bissextile year (equal to 4.35); only time points after the first vaccination and until 12 months from baseline were considered; ^(a)^ = only data on SAEs were reported; ^(b)^ = both data at 24 and 28 weeks were reported and only the data at 28 weeks were considered. Time-points regarding the Anal infections, Oral infections and High Grade Anal Intraepithelial Neoplasia were not reported in the Table since these outcomes were reported only in one study (Wilkin et al, 2018) and were related to the whole study period.
