# Supplementary material for: Efficacy and safety of human papillomavirus vaccination in HIV-infected patients: a systematic review and meta-analysis
Source: Sci Rep. 2021 Mar 2;11:4954. doi: 10.1038/s41598-021-83727-7 (PMC7925667; doi:10.1038/s41598-021-83727-7)
Supplement: Supplementary file 8 — SupplementaryTable S4 [file 41598_2021_83727_MOESM8_ESM.docx]

**Supplementary table 4.** Adverse events reported in the manuscripts or on ClinicalTrials.gov registry.

|  | Denny, 2013 | | Levin, 2010 | | Hidalgo-Tenorio, 2017 | | Wilkin, 2018 | |
| --- | --- | --- | --- | --- | --- | --- | --- | --- |
|  | Vaccine | Placebo | Vaccine | Placebo | Vaccine | Placebo | Vaccine | Placebo |
|  | N | N | N | N | N | N | N | N |
| **Safety outcomes considered in this review** | | | | | | | | |
| Serious adverse events | 3 | 2 | 7 | 2 | 0 | 0 | 8 | 23 |
| Not serious adverse events | 61 | 55 | 54 | 13 | 59 | 62 | - | - |
| All-Cause Mortality | 0 | 0 | - | - | 0 | 0 | 0 | 4 |
| **Adverse events after each vaccine dose** | | | | | | | | |
| Total AE, dose 1 | - | - | - | - | 36 | 55 | - | - |
| Total AE, dose 2 | - | - | - | - | 59 | 62 | - | - |
| Total AE, dose 3 | - | - | - | - | 44 | 57 | - | - |
| Serious AE, dose 1 | - | - | - | - | 0 | 0 | - | - |
| Serious AE, dose 2 | - | - | - | - | 0 | 0 | - | - |
| Serious AE, dose 3 | - | - | - | - | 0 | 0 | - | - |
| Deaths, dose 1 | - | - | - | - | 0 | 0 | - | - |
| Deaths, dose 2 | - | - | - | - | 0 | 0 | - | - |
| Deaths, dose 3 | - | - | - | - | 0 | 0 | - | - |
| Injection-site pain, dose 1 | - | - | - | - | 37 | 53 | - | - |
| Injection-site pain, dose 2 | - | - | - | - | 58 | 62 | - | - |
| Injection-site pain, dose 3 | - | - | - | - | 45 | 57 | - | - |
| Local itching, dose 1 | - | - | - | - | 10 | 5 | - | - |
| Local itching, dose 2 | - | - | - | - | 1 | 1 | - | - |
| Local itching, dose 3 | - | - | - | - | 1 | 0 | - | - |
| Syncope, dose 1 | - | - | - | - | 2 | 0 | - | - |
| Syncope, dose 1 | - | - | - | - | 1 | 0 | - | - |
| Syncope, dose 1 | - | - | - | - | 1 | 0 | - | - |
| **Serious Adverse Events (Total)** | | | | | | | | |
| Abdominal mass | - | - | - | - | - | - | 1 | 0 |
| Abdominal pain | - | - | - | - | - | - | 1 | 1 |
| Acute myocardial infarction | - | - | - | - | - | - | 0 | 2 |
| Acute psychosis | - | - | - | - | - | - | 1 | 0 |
| Acute respiratory failure | - | - | - | - | - | - | 1 | 0 |
| Alcohol withdrawal syndrome | - | - | - | - | - | - | 1 | 1 |
| Alveolitis allergic | - | - | - | - | - | - | 1 | 0 |
| Anal cancer | - | - | - | - | - | - | 1 | 1 |
| Anal fistula | - | - | - | - | - | - | 1 | 0 |
| Anal squamous cell carcinoma | - | - | - | - | - | - | 0 | 1 |
| Appendicitis | - | - | - | - | - | - | 1 | 0 |
| Asthma | - | - | - | - | - | - | 1 | 0 |
| B-cell lymphoma | - | - | - | - | - | - | 0 | 1 |
| Basal cell carcinoma | - | - | - | - | - | - | 1 | 1 |
| Blood amylase increased | 1 | 0 | 1 | 0 | - | - | - | - |
| Blood bilirubin increased | 1 | 0 | 1 | 0 | - | - | - | - |
| Blood bilirubin unconjugated increased | 0 | 1 | 0 | 1 | - | - | - | - |
| Bronchitis | - | - | - | - | - | - | 0 | 2 |
| Cellulitis | - | - | - | - | - | - | 1 | 0 |
| Cerebrovascular accident | - | - | - | - | - | - | 1 | 0 |
| Chest pain | - | - | - | - | - | - | 2 | 5 |
| Chlamydial infection | - | - | - | - | - | - | 1 | 0 |
| Cholelithiasis | - | - | - | - | - | - | 0 | 1 |
| Chronic obstructive pulmonary disease | - | - | - | - | - | - | 0 | 4 |
| Colitis | - | - | - | - | - | - | 1 | 0 |
| Completed suicide | - | - | - | - | - | - | 0 | 1 |
| Coronary artery disease | - | - | - | - | - | - | 0 | 1 |
| Dehydration | - | - | - | - | - | - | 0 | 1 |
| Depression | - | - | - | - | - | - | 1 | 0 |
| Diverticulitis | - | - | - | - | - | - | 0 | 2 |
| Dyspnoea | - | - | - | - | - | - | 0 | 1 |
| Fall | - | - | - | - | - | - | 0 | 1 |
| Follicle centre lymphoma diffuse small cell lymphoma | - | - | - | - | - | - | 0 | 1 |
| Foot fracture | - | - | - | - | - | - | 0 | 1 |
| Gastroenteritis viral | - | - | - | - | - | - | 1 | 1 |
| Gastroenteritis | - | - | - | - | - | - | 0 | 2 |
| Gastroenteritis | 1 | 0 | - | - | - | - | - | - |
| Gastrointestinal haemorrhage | - | - | - | - | - | - | 0 | 1 |
| Genital ulceration | - | - | - | - | - | - | 0 | 1 |
| Haemorrhagic stroke | - | - | - | - | - | - | 0 | 1 |
| Hodgkin's disease | - | - | - | - | - | - | 1 | 0 |
| Hypotension | - | - | - | - | - | - | 0 | 1 |
| Influenza | - | - | - | - | - | - | 2 | 2 |
| Intervertebral disc operation | - | - | - | - | - | - | 1 | 0 |
| Large intestine perforation | - | - | - | - | - | - | 0 | 1 |
| Lobar pneumonia | 0 | 1 | - | - | - | - | - | - |
| Lower limb fracture | - | - | - | - | - | - | 1 | 0 |
| Meningitis viral | - | - | - | - | - | - | 1 | 0 |
| Mental status changes | - | - | - | - | - | - | 0 | 1 |
| Migraine | 1 | 0 | - | - | - | - | - | - |
| Multiple injuries | - | - | - | - | - | - | 1 | 0 |
| Myocardial infarction | - | - | - | - | - | - | 0 | 1 |
| Neutropenia | 2 | 0 | 2 | 0 | - | - | - | - |
| Neutrophil count decreased | 1 | 0 | 1 | 0 | - | - | - | - |
| Oedema peripheral | 0 | 1 | 0 | 1 | - | - | - | - |
| Oesophageal adenocarcinoma | - | - | - | - | - | - | 0 | 1 |
| Orchitis | - | - | - | - | - | - | 0 | 1 |
| Osteoarthritis | - | - | - | - | - | - | 0 | 1 |
| Overdose | - | - | - | - | - | - | 0 | 1 |
| Pancreatic carcinoma metastatic | - | - | - | - | - | - | 0 | 1 |
| Pancreatitis acute | - | - | - | - | - | - | 0 | 2 |
| Pancreatitis chronic | - | - | - | - | - | - | 0 | 1 |
| Pancreatitis | - | - | - | - | - | - | 0 | 1 |
| Pericardial effusion | - | - | - | - | - | - | 1 | 0 |
| Perirectal abscess | - | - | - | - | - | - | 0 | 1 |
| Peritonsillar abscess | - | - | - | - | - | - | 1 | 0 |
| Pleural effusion | - | - | - | - | - | - | 1 | 1 |
| Pneumonia bacterial | 1 | 1 | - | - | - | - | - | - |
| Pneumonia pneumococcal | - | - | - | - | - | - | 1 | 0 |
| Pneumonia streptococcal | - | - | - | - | - | - | 0 | 1 |
| Pneumonia | - | - | - | - | - | - | 2 | 3 |
| Primary syphilis | - | - | - | - | - | - | 0 | 1 |
| Prostate cancer | - | - | - | - | - | - | 1 | 1 |
| Pseudomembranous colitis | - | - | - | - | - | - | 1 | 0 |
| Psychotic disorder | 0 | 0 | - | - | - | - | 0 | 1 |
| Pulmonary hypertension | - | - | - | - | - | - | 0 | 1 |
| Pyelonephritis | - | - | - | - | - | - | 0 | 1 |
| Pyrexia | - | - | - | - | - | - | 0 | 1 |
| Radius fracture | - | - | - | - | - | - | 0 | 1 |
| Renal cell carcinoma | - | - | - | - | - | - | 0 | 1 |
| Road traffic accident | - | - | - | - | - | - | 0 | 1 |
| Scrotal abscess | - | - | - | - | - | - | 0 | 1 |
| Seizure | - | - | - | - | - | - | 1 | 0 |
| Sepsis | - | - | - | - | - | - | 1 | 3 |
| Skull fracture | 0 | 1 | - | - | - | - | - | - |
| Small intestinal obstruction | - | - | - | - | - | - | 0 | 3 |
| Squamous cell carcinoma of head and neck | - | - | - | - | - | - | 0 | 1 |
| Stab wound | - | - | - | - | - | - | 1 | 0 |
| Substance abuse | - | - | - | - | - | - | 0 | 1 |
| Suicide attempt | 0 | 0 | - | - | - | - | 1 | 1 |
| Syncope | - | - | - | - | - | - | 0 | 2 |
| Transitional cell carcinoma | - | - | - | - | - | - | 1 | 0 |
| Viral infection | - | - | - | - | - | - | 0 | 1 |
| Weight decreased | - | - | - | - | - | - | 0 | 1 |
| **Not Serious Adverse Events (Total)** | | | | | | | | |
| Abdominal pain | 2 | 2 | 7 | 0 | - | - | 13 | 16 |
| Acne | 1 | 0 | - | - | - | - | - | - |
| Acute stress disorder | 1 | 0 | - | - | - | - | - | - |
| Alanine aminotransferase increased | - | - | 20 | 6 | - | - | - | - |
| Anaemia | 2 | 0 | - | - | - | - | - | - |
| Anogenital dysplasia | - | - | - | - | - | - | 11 | 20 |
| Arthralgia | 4 | 5 | - | - | - | - | 14 | 18 |
| Arthropod bite | 0 | 1 | - | - | - | - | - | - |
| Aspartate aminotransferase increased | - | - | 15 | 10 | - | - | - | - |
| Asthma | - | - | 3 | 4 | - | - | - | - |
| Axillary mass | 1 | 0 | - | - | - | - | - | - |
| Back pain | 5 | 4 | - | - | - | - | - | - |
| Bartholin’s abscess | 0 | 1 | - | - | - | - | - | - |
| Blood albumin abnormal | - | - | 3 | 2 | - | - | - | - |
| Blood alkaline phosphatase increased | - | - | 14 | 1 | - | - | - | - |
| Blood bicarbonate abnormal | - | - | 21 | 5 | - | - | - | - |
| Blood bilirubin increased | - | - | 11 | 6 | - | - | - | - |
| Blood cholesterol increased | - | - | 17 | 4 | - | - | - | - |
| Blood creatinine increased | - | - | 5 | 3 | - | - | - | - |
| Blood glucose decreased | - | - | 37 | 10 | - | - | - | - |
| Blood glucose increased | - | - | 12 | 7 | - | - | - | - |
| Blood phosphorus decreased | - | - | 7 | 1 | - | - | - | - |
| Blood potassium decreased | - | - | 12 | 3 | - | - | - | - |
| Blood sodium decreased | - | - | 28 | 11 | - | - | - | - |
| Blood triglycerides increased | - | - | 7 | 0 | - | - | - | - |
| Breast pain | 1 | 0 | - | - | - | - | - | - |
| Bronchial hyperreactivity | - | - | 1 | 2 | - | - | - | - |
| Bronchitis | 2 | 1 | - | - | - | - | - | - |
| Bronchospas | 1 | 0 | - | - | - | - | - | - |
| Candidiasis | 0 | 0 | - | - | - | - | - | - |
| CD4 lymphocytes decreased | 2 | 1 | - | - | - | - | - | - |
| Cellulitis | 0 | 0 | - | - | - | - | - | - |
| Chest pain | - | - | 5 | 0 | - | - | - | - |
| Chills | - | - | - | - | - | - | 21 | 17 |
| Conjunctivitis | 1 | 1 | 5 | 0 | - | - | - | - |
| Cough | 8 | 5 | 13 | 9 | - | - | 33 | 22 |
| Dermatitis allergic | 0 | 1 | - | - | - | - | - | - |
| Dermatitis contact | 0 | 1 | - | - | - | - | - | - |
| Diarrhoea | 4 | 6 | 7 | 2 | - | - | 16 | 26 |
| Dizziness | 4 | 4 | - | - | - | - | - | - |
| Dysmenorrhoea | 1 | 1 | - | - | - | - | - | - |
| Dyspepsia | 1 | 1 | - | - | - | - | - | - |
| Dyspnoea | - | - | - | - | - | - | 19 | 20 |
| Dysuria | 0 | 0 | - | - | - | - | - | - |
| Ear pain | 1 | 2 | 5 | 3 | - | - | - | - |
| Eczema | 1 | 0 | - | - | - | - | - | - |
| Eosinophilia | 0 | 1 | - | - | - | - | - | - |
| Epistaxis | 0 | 3 | - | - | - | - | - | - |
| Erythema | 0 | 0 | - | - | - | - | - | - |
| Eye swelling | 0 | 1 | - | - | - | - | - | - |
| Face injury | 1 | 0 | - | - | - | - | - | - |
| Fatigue | 18 | 16 | - | - | - | - | 18 | 19 |
| Flank pain | 0 | 1 | - | - | - | - | - | - |
| Folliculitis | 0 | 0 | - | - | - | - | - | - |
| Gait disturbance | - | - | 0 | 2 | - | - | - | - |
| Gastritis | 1 | 1 | - | - | - | - | - | - |
| Gastroenteritis viral | 0 | 0 | - | - | - | - | - | - |
| Gastroenteritis | 1 | 0 | 7 | 0 | - | - | - | - |
| Gastrointestinal disorder | 15 | 11 | - | - | - | - | - | - |
| Haemoglobin decreased | - | - | 5 | 1 | - | - | - | - |
| Headache | 31 | 33 | 6 | 2 | - | - | 16 | 17 |
| Herpes simplex | 0 | 2 | - | - | - | - | - | - |
| Herpes virus infection | 1 | 0 | - | - | - | - | - | - |
| Herpes zoster | 1 | 1 | - | - | - | - | - | - |
| Hordeolum | 0 | 0 | - | - | - | - | - | - |
| Influenza like illness | 1 | 1 | - | - | - | - | - | - |
| Influenza | 1 | 1 | - | - | - | - | - | - |
| Injection site erythema | 7 | 1 | - | - | - | - | - | - |
| Injection site induration | 4 | 0 | - | - | - | - | - | - |
| Injection site mass | 3 | 0 | - | - | - | - | - | - |
| Injection site pruritus | 3 | 0 | - | - | - | - | - | - |
| Insomnia | 0 | 1 | - | - | - | - | - | - |
| Joint injury | 0 | 1 | - | - | - | - | - | - |
| Measles | 2 | 0 | - | - | - | - | - | - |
| Menorrhagia | 0 | 2 | - | - | - | - | - | - |
| Metrorrhagia | 1 | 0 | - | - | - | - | - | - |
| Muscle spasms | 0 | 0 | - | - | - | - | - | - |
| Muscular weakness | 0 | 1 | - | - | - | - | - | - |
| Musculoskeletal pain | 1 | 0 | - | - | - | - | - | - |
| Myalgia | 4 | 3 | - | - | - | - | - | - |
| Nasal obstruction/Nasal congestion | 2 | 0 | 10 | 6 | - | - | 18 | 14 |
| Nasopharyngitis | 3 | 6 | - | - | - | - | - | - |
| Nausea | 1 | 0 | - | - | - | - | 16 | 20 |
| Neck pain | 0 | 1 | - | - | - | - | - | - |
| Neutropenia | 2 | 0 | - | - | - | - | - | - |
| Neutrophil count decreased | 1 | 1 | 36 | 10 | - | - | - | - |
| Night sweats | 0 | 1 | - | - | - | - | - | - |
| Oedema peripheral | 0 | 1 | - | - | - | - | - | - |
| Oral herpes | 1 | 1 | - | - | - | - | - | - |
| Oropharyngeal pain | 2 | 1 | 11 | 6 | - | - | 19 | 10 |
| Otitis media/Otitis media acute | 0 | 1 | 4 | 3 | - | - | - | - |
| Pain in extremity | 3 | 2 | 22 | 19 | - | - | - | - |
| Pain | 52 | 27 | - | - | - | - | - | - |
| Paraesthesia | 0 | 1 | - | - | - | - | - | - |
| Paronychia | 0 | 0 | - | - | - | - | - | - |
| Peptic ulcer | 0 | 0 | - | - | - | - | - | - |
| Pharyngitis streptococcal | - | - | 3 | 3 | - | - | - | - |
| Pharyngitis | 1 | 2 | 5 | 3 | - | - | - | - |
| Platelet count decreased | - | - | 2 | 2 | - | - | - | - |
| Pneumonia | - | - | 2 | 2 | - | - | - | - |
| Pruritus generalised | 1 | 0 | - | - | - | - | - | - |
| Purulent discharge | - | - | 0 | 2 | - | - | - | - |
| Pyrexia | 2 | 3 | 14 | 10 | - | - | 0 | 1 |
| Rash pruritic | 0 | 1 | - | - | - | - | - | - |
| Rash | 3 | 2 | 5 | 2 | - | - | - | - |
| Rhinitis allergic | 0 | 1 | - | - | - | - | - | - |
| Rhinitis seasonal | 1 | 1 | - | - | - | - | - | - |
| Rhinitis | 3 | 1 | - | - | - | - | - | - |
| Rhonchi | - | - | 0 | 2 | - | - | - | - |
| Sexually transmitted disease | 0 | 1 | - | - | - | - | - | - |
| Sinusitis/Acute sinusitis | 1 | 1 | 4 | 5 | - | - | - | - |
| Skin lesion | - | - | 0 | 2 | - | - | - | - |
| Stab wound | 0 | 1 | - | - | - | - | - | - |
| Subcutaneous abscess | 1 | 1 | - | - | - | - | - | - |
| Swelling | 19 | 2 | - | - | - | - | - | - |
| Thermal burn | 1 | 0 | - | - | - | - | - | - |
| Throat irritation | 0 | 1 | - | - | - | - | - | - |
| Tinea infection | 1 | 0 | - | - | - | - | - | - |
| Tonsillitis | 0 | 0 | - | - | - | - | - | - |
| Tooth abscess | 0 | 1 | - | - | - | - | - | - |
| Tooth extraction | 1 | 1 | - | - | - | - | - | - |
| Toothache | 0 | 0 | - | - | - | - | - | - |
| Upper respiratory tract infection | 10 | 10 | - | - | - | - | - | - |
| Urinary tract infection | 1 | 2 | - | - | - | - | - | - |
| Vaginitis bacterial | 0 | 1 | - | - | - | - | - | - |
| Varicella | 1 | 0 | - | - | - | - | - | - |
| Vessel puncture site pain/Injection site pain | 2 | 0 | 13 | 2 | - | - | - | - |
| Viral pharyngitis | 0 | 1 | - | - | - | - | - | - |
| Vomiting | 0 | 1 | 9 | 4 | - | - | 7 | 20 |
| Vulvovaginal candidiasis | 5 | 4 | - | - | - | - | - | - |
| Vulvovaginal human papilloma virus infection | 1 | 0 | - | - | - | - | - | - |
| Vulvovaginal pruritus | 1 | 2 | - | - | - | - | - | - |
| Vulvovaginitis trichomonal | 0 | 1 | - | - | - | - | - | - |
| Wheezing | - | - | 5 | 5 | - | - | - | - |
